# Lipid deposition promotes YTHDF3-mediated m^6^A modification of PPARα to facilitate liver metastasis of colorectal cancer

**DOI:** 10.1093/procel/pwaf092

**Published:** 2025-11-05

**Authors:** Wen Ni, Yuanyuan Xu, Mengrou Zhang, Yuqing Li, Piao Huang, Zhun Li, Qi Wu, Hui Mo, Yibiao Ye, Yuhui Li, Aijun Zhou, Su Yao, Shilin Zhi, Jiali Qi, Shuhui Yu, Saiqi He, Jianming Li

**Affiliations:** Department of Pathology, Sun Yat-sen Memorial Hospital, Sun Yat-sen University, Guangzhou 510120, China; Guangdong Provincial Key Laboratory of Malignant Tumor Epigenetics and Gene Regulation, Sun Yat-sen Memorial Hospital, Sun Yat-sen University, Guangzhou 510120, China; Department of Pathology, Sun Yat-sen Memorial Hospital, Sun Yat-sen University, Guangzhou 510120, China; Guangdong Provincial Key Laboratory of Malignant Tumor Epigenetics and Gene Regulation, Sun Yat-sen Memorial Hospital, Sun Yat-sen University, Guangzhou 510120, China; Department of Pathology, Sun Yat-sen Memorial Hospital, Sun Yat-sen University, Guangzhou 510120, China; Guangdong Provincial Key Laboratory of Malignant Tumor Epigenetics and Gene Regulation, Sun Yat-sen Memorial Hospital, Sun Yat-sen University, Guangzhou 510120, China; Department of Pathology, Sun Yat-sen Memorial Hospital, Sun Yat-sen University, Guangzhou 510120, China; Guangdong Provincial Key Laboratory of Malignant Tumor Epigenetics and Gene Regulation, Sun Yat-sen Memorial Hospital, Sun Yat-sen University, Guangzhou 510120, China; Department of Pathology, Sun Yat-sen Memorial Hospital, Sun Yat-sen University, Guangzhou 510120, China; Guangdong Provincial Key Laboratory of Malignant Tumor Epigenetics and Gene Regulation, Sun Yat-sen Memorial Hospital, Sun Yat-sen University, Guangzhou 510120, China; Department of Pathology, Sun Yat-sen Memorial Hospital, Sun Yat-sen University, Guangzhou 510120, China; Guangdong Provincial Key Laboratory of Malignant Tumor Epigenetics and Gene Regulation, Sun Yat-sen Memorial Hospital, Sun Yat-sen University, Guangzhou 510120, China; Department of Pathology, Sun Yat-sen Memorial Hospital, Sun Yat-sen University, Guangzhou 510120, China; Guangdong Provincial Key Laboratory of Malignant Tumor Epigenetics and Gene Regulation, Sun Yat-sen Memorial Hospital, Sun Yat-sen University, Guangzhou 510120, China; Department of Pathology, Sun Yat-sen Memorial Hospital, Sun Yat-sen University, Guangzhou 510120, China; Guangdong Provincial Key Laboratory of Malignant Tumor Epigenetics and Gene Regulation, Sun Yat-sen Memorial Hospital, Sun Yat-sen University, Guangzhou 510120, China; Department of Hepato-Billiary Surgery, Sun Yat-sen Memorial Hospital, Sun Yat-sen University, Guangzhou 510120, China; Department of Pathology, Sun Yat-sen Memorial Hospital, Sun Yat-sen University, Guangzhou 510120, China; Guangdong Provincial Key Laboratory of Malignant Tumor Epigenetics and Gene Regulation, Sun Yat-sen Memorial Hospital, Sun Yat-sen University, Guangzhou 510120, China; Department of Pathology, Sun Yat-sen Memorial Hospital, Sun Yat-sen University, Guangzhou 510120, China; Department of Pathology, Guangdong Provincial People’s Hospital, Guangdong Academy of Medical Sciences, Southern Medical University, Guangzhou 510080, China; Department of Pathology, Sun Yat-sen Memorial Hospital, Sun Yat-sen University, Guangzhou 510120, China; Guangdong Provincial Key Laboratory of Malignant Tumor Epigenetics and Gene Regulation, Sun Yat-sen Memorial Hospital, Sun Yat-sen University, Guangzhou 510120, China; Department of Pathology, Sun Yat-sen Memorial Hospital, Sun Yat-sen University, Guangzhou 510120, China; Guangdong Provincial Key Laboratory of Malignant Tumor Epigenetics and Gene Regulation, Sun Yat-sen Memorial Hospital, Sun Yat-sen University, Guangzhou 510120, China; Department of Pathology, Sun Yat-sen Memorial Hospital, Sun Yat-sen University, Guangzhou 510120, China; Guangdong Provincial Key Laboratory of Malignant Tumor Epigenetics and Gene Regulation, Sun Yat-sen Memorial Hospital, Sun Yat-sen University, Guangzhou 510120, China; Department of Pathology, Sun Yat-sen Memorial Hospital, Sun Yat-sen University, Guangzhou 510120, China; Guangdong Provincial Key Laboratory of Malignant Tumor Epigenetics and Gene Regulation, Sun Yat-sen Memorial Hospital, Sun Yat-sen University, Guangzhou 510120, China; Department of Pathology, Sun Yat-sen Memorial Hospital, Sun Yat-sen University, Guangzhou 510120, China; Guangdong Provincial Key Laboratory of Malignant Tumor Epigenetics and Gene Regulation, Sun Yat-sen Memorial Hospital, Sun Yat-sen University, Guangzhou 510120, China; The MOE Basic Research and Innovation Center for the Targeted Therapeutics of Solid Tumors, The First Affiliated Hospital, Jiangxi Medical College, Nanchang University, Nanchang 330006, China; Department of Pathology and Institute of Molecular Pathology, The First Affiliated Hospital, Jiangxi Medical College, Nanchang University, Nanchang 330006, China

**Keywords:** colorectal cancer, lipid metabolism reprogramming, m^6^A modification, β-hydroxybutyrylation, liver metastasis, metabolites

## Abstract

The liver is a common site for cancer metastasis and a key metabolic organ. Lipid metabolism irregularities are linked to liver metastasis risk, but the mechanisms are not fully understood. Herein, in colorectal cancer liver metastasis (CRLM) clinical samples, lipid metabolism was broadly dysregulated, and lipid metabolites accumulated, as shown by integrated transcriptome and lipidomics analyses. Functionally, lipid deposition promotes liver metastasis *in vitro* and *in vivo*. Mechanistically, lipid deposition significantly enhances YTHDF3-mediated m^6^A modification and degradation of PPARα, which is crucial for liver metastasis. This process reduces the β-hydroxybutyrylation of YTHDF3, thereby promoting LLPS and increasing the stability of YTHDF3, which in turn facilitates the progression of CRC and liver metastasis. Furthermore, lipid deposition induces the interaction between STAT3 and YAP, activating YTHDF3 transcription. These two regulatory mechanisms synergize to drive YTHDF3 accumulation in lipid-rich metastatic lesions. In summary, our findings reveal that lipid deposition promotes LLPS-mediated m^6^A modification and decreases β-hydroxybutyrylation in liver metastasis, offering new strategies for the treatment of CRLM.

## Introduction

Colorectal cancer (CRC) ranks as one of the second most prevalent malignant tumors in China, and colorectal cancer liver metastasis (CRLM) constitutes the principal cause of death for patients with CRC (Han *et al.*, 2024; Steeg, 2006; Tsilimigras *et al.*, 2021). Approximately 15%–25% of patients diagnosed with CRC present with liver metastases, while another 15%–25% develop liver metastases subsequent to the radical resection of the primary CRC. Among them, the vast majority of liver metastases are initially non-resectable radically (Adam and Vinet, 2004; de Jong *et al.*, 2009; Vibert *et al.*, 2005). The study of CRLM confronts significant challenges, one of which is mainly due to the difficulty in obtaining pathological samples of paired liver metastases that have not undergone chemoradiotherapy.

Lipid metabolic reprogramming is crucial for tumor ­progression, providing the necessary materials for tumor cells to maintain viability and synthesize new biomass (Broadfield *et al.*, 2021; Corn *et al.*, 2020). Unlike normal cells, tumor cells actively synthesize and accumulate substantial lipid quantities to fuel rapid tumor growth. They also store surplus lipids in lipid droplets (LDs) as an energy reserve for times of metabolic stress (Meng *et al.*, 2024). Abnormal lipid metabolism has been recognized as a substantial risk factor for both liver diseases and liver metastasis of cancers (Earl *et al.*, 2009; Loomba *et al.*, 2021; Ocak Duran *et al.*, 2015; Ohashi *et al.*, 2019; Shen *et al.*, 2010; VanSaun *et al.*, 2009; Yoshimoto *et al.*, 2013). A longitudinal cohort study indicated that nonalcoholic fatty liver disease (NAFLD) was correlated with an augmented risk of gastrointestinal cancers (Allen *et al.*, 2019). Another prospective cohort study regarded hepatic steatosis as an independent risk factor of liver recurrence after resection of colorectal liver metastases (Hamady *et al.*, 2013). The hepatocyte-derived extracellular vesicles in fatty liver could facilitate a metastatic tumor microenvironment for CRC liver metastasis (Wang *et al.*, 2023). Nevertheless, the lipid metabolic profile of CRLM has not been examined in detail, and the underlying mechanisms remain poorly comprehended.

N^6^-methyladenosine (m^6^A) modification is intricately linked to the reprogramming of cellular metabolism of tumors. It is sensitive to environmental changes such as nutrient availability, to alter the transcriptome in response to these changes, leading to the reprogramming of metabolic pathways to adapt to new conditions (Salisbury *et al.*, 2021; Weng *et al.*, 2022). METTL14 is reported inversely correlated with fatty liver disease by triggering lipogenic transcripts for degradation and guards against liver lipid accumulation (Salisbury *et al.*, 2021). Interestingly, we found that YTHDF3 (YTH domain-containing family protein 3), readers of m^6^A modification, mediated phosphofructokinase PFKL expression in glycolysis in hepatocellular carcinoma progression (Zhou *et al.*, 2022). However, the function and regulation of m^6^A modification in liver metastasis remains underway.

Liquid–liquid phase separation (LLPS) is a physicochemical process through which proteins and/or nucleic acids demix from the surrounding environment to form membrane-less, highly dynamic condensates (e.g., stress granules or nuclear speckles). LLPS compartmentalizes biochemical reactions, influences chromatin organization, transcription, and signal transduction, and has recently emerged as a reversible driver of tumor initiation and progression (Alberti and Dormann, 2019; Li *et al.*, 2025; Mehta and Zhang, 2022; Tong *et al.*, 2022; Zhao and Zhang, 2020). NUP98 fusion oncoproteins undergo LLPS to form nuclear puncta, which are critical for leukemogenesis (Chandra *et al.*, 2022). YBX1 could undergo LLPS and sort miR-223 into exosomes and secreted from cells (Liu *et al.*, 2021). The mRNA-YTHDF complexes undergo LLPS, which is subject to compartment-specific regulation, including reduced mRNA stability and translation (Ries *et al.*, 2019). A recent study showed glycogen accumulation is reported as a key regulator of LLPS in liver tumor initiation (Liu *et al.*, 2021). However, the link between lipogenesis and LLPS was unknown, and whether LLPS contributes to the liver metastasis of tumors remains unclear.

The altered metabolites could influence proteins’ post-translational modification, such as lactylation (Zhang *et al.*, 2019), succinylation (Yang and Gibson, 2019), crotonylation (Yuan *et al.*, 2023), and hydroxybutyrylation (Koronowski *et al.*, 2021; Xie *et al.*, 2016). Lysine β-hydroxybutyrylation (Kbhb) modification is a new, β-hydroxybutyrate (BHB)-derived acylation reaction, which is strongly involved in many biological processes such as metabolic cardiovascular diseases (Shimazu *et al.*, 2013), neuropsychiatric disorders (Kashiwaya *et al.*, 2000), and tumors (Liu *et al.*, 2019; Zhang *et al.*, 2021). BHB is the main component of ketone bodies, which is mainly synthesized through fatty acid β-oxidation (FAO) in the liver (Dedkova and Blatter, 2014). FAO serves as a key metabolic process responsible for the degradation of fatty acids, especially when glucose levels are low, as occurs during fasting periods (Du *et al.*, 2024; Houten *et al.*, 2016). However, the impact and mechanism of BHB and Kbhb modification in tumor metastasis remain largely unknown.

Herein, we demonstrate that the lipid metabolic reprogramming is modulated by YTHDF3-mediated m^6^A modification, which promotes CRLM. We discover that lipid accumulation is anomalously upregulated in CRLM, which significantly promotes CRC cell proliferation and invasion. Further investigations have uncovered that lipid deposition promotes YTHDF3-mediated m^6^A modification and degradation of PPARα (peroxisome proliferator-activated receptor alpha). This process alleviates the β-hydroxybutyrylation of YTHDF3, thereby fostering LLPS and increased stability of YTHDF3, which promotes CRC progression and liver metastasis. Collectively, these results disclose that YTHDF3-mediated m^6^A modification is accountable for the lipid metabolic reprogramming of CRLM. Our findings offer a promising clinical target for CRLM treatment and illuminate the mechanisms of liver metastasis and lipid metabolic reprogramming in CRC.

## Results

### Integrative transcriptome and lipidomic analysis profiling lipid metabolic reprogramming of CRLM

**Figure 1.**
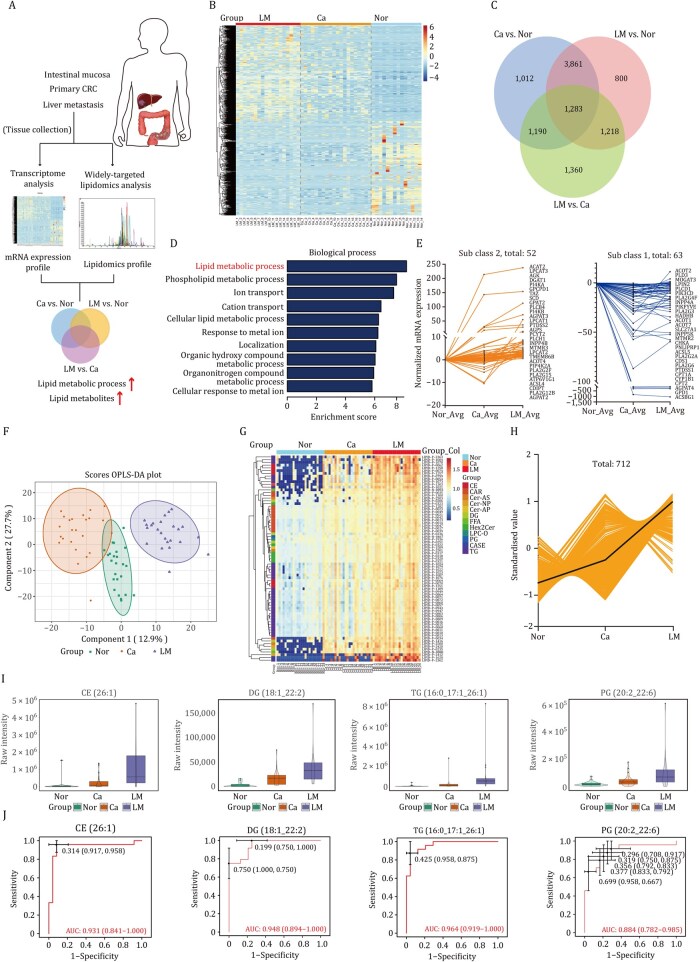
**Integrative transcriptome and lipidomic analysis profiling lipid metabolic reprogramming of CRLM**. (A) Schematic workflow for lipidomics and transcriptome profiling of normal intestinal mucosal tissues (Nor), primary tumors (Ca), and paired liver metastasis lesions (LM) in CRC patients (*N *= 20). (B) Heatmap of transcriptome profiling among Nor, Ca, and LM. (C) The overlapping region of the Venn diagram represents genes that are consistently dysregulated in both primary colorectal cancer and liver metastasis, relative to the matched normal mucosa. (D) Intersection analysis of GO analysis for differentially expressed genes in Nor, Ca, and LM (fold change ≥2; *P*-value < 0.05 in RNA-seq). (E) STEM analysis to identify differentially expressed genes involved in lipolysis and lipogenesis pathways. (F) OPLS-DA of the lipidomics data from Nor, Ca, and LM (*n *= 24). (G) Heatmap of significantly differential lipid metabolites among Nor, Ca, and LM (fold change ≥2; *P*-value < 0.05). (H) K-means clustering of differential lipid metabolites among Nor, Ca, and LM. Two-sided Kruskal–Wallis tests followed by Benjamini–Hochberg (BH) multiple comparison test with FDR < 0.1. (I) Volcano plots of the significantly differential metabolites in Nor, Ca, and LM. (J) The receiver operating characteristic (ROC) curve of cholesteryl ester [CE (26:1)], diacylglycerol [DG (18:1_22:2)], phosphatidylglycerol [PG (20:2_22:6)], and triacylglycerol [TG (16:0_17:1_26:1)]. AUC, area under the curve. A two-sided Wilcoxon rank-sum test was used.

We carried out transcriptome sequencing and lipidomic analysis on normal intestinal mucosal tissues, as well as on the matched primary CRC tissues and liver metastases lesions from 20 participants, with the aim of characterizing differential lipid metabolites and the key factors in CRLM (Fig. 1A). Advanced heatmap and Venn diagram analysis to detect differentially expressed genes that are consistently dysregulated in both primary CRC and liver metastasis relative to the matched normal mucosa (Fig. 1B and 1C). Gene ontology and the Short Time - series Expression Miner (STEM) analysis showed that genes related to lipid anabolism were greatly upregulated, and lipid catabolism was broadly declined in tumor tissues and liver metastasis lesions compared with tumor-adjacent mucosa (Fig. 1D and 1E). Widely-targeted lipidomics analysis revealed remarkable lipid metabolite accumulation in liver metastasis lesions. Heatmap and K-means clustering analysis identified that lipid metabolites were consistently upregulated in liver metastasis lesions (Fig. 1G and 1H). Through mass spectrum-based feature selection, orthogonal partial least-squares discriminant analysis (OPLS-DA) was used to supervise the group difference (Fig. 1F). We found that lipid metabolites of glyceride, cholesterol ester, and glycerophospholipids were broadly upregulated in CRLM. And the differential lipid metabolite profiles have been displayed in Tables S1 and S5 and Figs S1–3. Among the most severely changed metabolites in liver metastasis lesions, we identified lipids metabolites such as cholesterol ester 26:1, diglyceride 18:1_22:2, phosphatidyl glycerol 20:2_22:6, and triglyceride 16:0_17:1_26:1 is significant accumulated in CRLM (Fig. 1I). The AUC (area under the curve) values of these metabolites achieved nearly 90%, and more than 90.00% sensitivity and specificity (Fig. 1J). In brief, lipid metabolites are anomalously upregulated in CRLM based on integrative transcriptome and lipidomic analysis.

### Lipid deposition promotes tumor proliferation and invasion *in vitro* and *in vivo*

**Figure 2.**
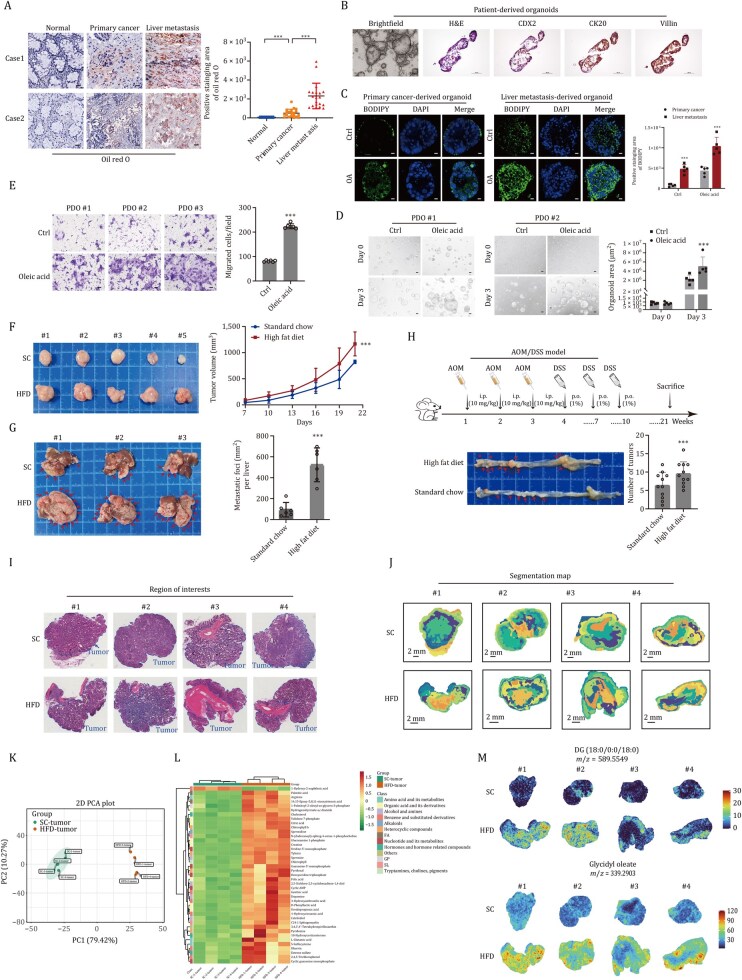
**Lipid deposition promotes tumor progression and liver metastasis *in vitro* and *in vivo***. (A) Oil red O staining of lipid deposition in Nor, Ca, and LM. The ruler represents a scale of 25 µm. ***P < 0.001. (B) IHC staining of PDOs of CRC tumors. The ruler represents a scale of 25 µm. (C) Lipids staining by BODIPY 493/503 in the PDOs of primary tumor and liver metastases. The ruler represents a scale of 25 µm. ***P < 0.001. (D and E) OA enhanced the proliferation (D) and invasion (E) potential of PDOs *in vitro*. The ruler represents a scale of 25 µm. ***P < 0.001. (F and G) Subcutaneous xenograft model (*n *= 5). (F) and mice splenic injection of liver metastases model (*n *= 6). (G) indicated that WD enhances CRC tumor cell progression and liver metastasis. ***P < 0.001 (*n *= 11). (H) AOM and DSS inducible model of colon carcinoma analysis showed that HFD enhances CRC tumor initiation and progression. ***P < 0.001. (I) The dashed blue line identified the substance of primary CRC tissues. (J) Segmentation map of spatial metabolomics visualized the differentially expressed pattern of metabolites in tumor tissues *in situ*. (K and L) PCA (K) and heatmap plots (L) indicated differentially expressed metabolites in the indicated groups. (M) The spatial distribution of differentially expressed metabolites of tumor tissues in the indicated groups.

LDs are essential cellular organelles for lipid storage, primarily composed of triglycerides and cholesterol esters, and are enveloped by a monolayer of phospholipids. Oil red O staining revealed consistent accumulation of LDs in the liver metastases of CRC (Fig. 2A). BODIPY 493/503 was used to visualize the neutral lipids of patient-derived organoids (PDOs) in primary tumor and liver metastases (Fig. 2B and 2C). Further *in vitro* assay identified that both oleic acid (OA) and palmitic acid (PA) promote the accumulation of intracellular LDs in cells, enhancing the proliferation and invasion potential of PDOs (Figs. 2C–E and S4A–E).

High-fat diet (HFD) feeds refer to a general category of feeds that is defined by a high intake of saturated fats (21% fat and 1.5% cholesterol). It has been widely used in the establishment of a variety of models, such as obesity, NAFLD, and metabolic-associated cancer. We performed a subcutaneous xenograft model and splenic injection of liver metastasis model in mice, achieving the results that HFD feed enhances CRC tumor progression and liver metastasis of tumor *in vivo* compared with standard chow (SC) (Fig. 2F and 2G).

We further established azoxymethane (AOM) and dextran sulfate sodium (DSS) inducible model of colon carcinoma, and the analysis showed that HFD feed enhances CRC tumor progression and lipid deposition (Fig. 2H). The spatial metabolomics was performed to identify the differentially expressed lipid metabolites in tumor tissues *in situ* (Fig. 2I and 2J). Principal-component analysis (PCA) and heatmap plots indicated that lipid metabolites in the western-diet (WD) group were greatly upregulated (Fig. 2K and 2L), and details differential metabolite profile was summarized in Table S2. The spatial distribution of differentially metabolites in tumor tissues of the indicated groups was shown in Fig. 2M. Further functional assays showed that lipid accumulation significantly promotes CRC cell proliferation and invasion, whereas C75, an inhibitor of lipid accumulation, yields the opposite results *in vitro* (Fig. S4F–I). As time-restricted feeding (TRF) has been shown to decrease and reverse fat accumulation in non-canonical fat-laden organs associated with obesity. We constructed TRF model in mice, and the results showed that TRF model inhibits tumor cell proliferation, liver metastasis, and lipid deposition *in vivo* (Fig. S4J–L). Overall, these findings indicate that excessive lipid deposition promotes tumor progression and liver metastasis *in vitro* and *in vivo*.

### Lipid deposition enhanced YTHDF3 expression

**Figure 3.**
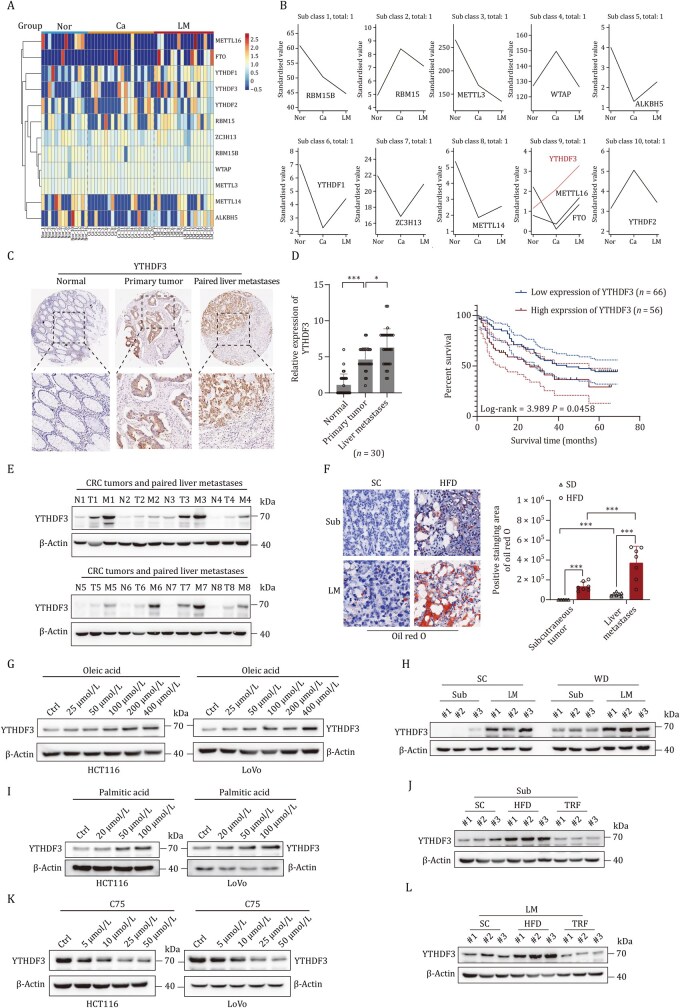
**Lipid accumulation enhances YTHDF3 expression**. (A) Transcriptome analysis identifies differentially expressed m^6^A regulators among Nor, Ca, and paired LM in liver metastasis of CRC. (B) STEM analysis reveals statistically significant expression profiles and genes involved in CRC liver metastasis. (C) Tissue microarray analysis of YTHDF3 expression levels in Nor, Ca, and LM of CRC patients. IHC scores for YTHDF3 are displayed in the boxplot using GraphPad Prism. ***P < 0.001, *P < 0.05. (D) Kaplan–Meier analysis of overall survival in primary CRC tumors stratified by YTHDF3 protein expression (*n *= 122). “Higher YTHDF3” refers to the upper 50% of IHC staining scores in primary tumors; “lower YTHDF3” refers to the lower 50%. (E) Western blot shows YTHDF3 expression in Nor, Ca, and LM of CRC patients. (F, H, and K) Oil red O staining (F) and Western blots (H and K) of the indicated groups. (G, I, and J) Western blot analysis demonstrated the expression levels of YTHDF3 in CRC cells treated with OA (G), PA (I), or C75 (J).

RNA m^6^A modification contributes to the reprogramming of lipid metabolism. However, the function of m^6^A regulators in lipid metabolism of CRLM remains unclear. Our transcriptome analysis showed the expression of m^6^A regulators were broadly altered in primary tumor and liver metastases, such as Wilms tumor 1-associated protein (WTAP), methyltrans ferase-like 14 (METTL14), METTL3, METTL16, RNA-binding motif protein 15 (RBM15), zinc finger CCCH-type containing 13 (ZC3H13), fat mass and obesity-associated protein (FTO), alkB homologue 5 (ALKBH5), YTHDF1, YTHDF2 and YTHDF3 (Fig. 3A). Interestingly, STEM analysis indicated that among all these genes, YTHDF3 was the only consistently upregulated gene both in primary CRC tissues and liver metastases compared with normal intestinal mucosal tissues (Fig. 3B). And the protein level of YTHDF3 was consistently upregulated both in primary tumor and liver metastases compared with normal intestinal mucosal tissues in CRC patients (Fig. 3E).

We performed immunohistochemical (IHC) analyses in CRC tissues, which included 122 cases of CRC patients with clinical follow-up data collected at Sun Yat-sen Memorial Hospital. Results showed that the protein level of YTHDF3 was significantly increased in tumors and liver metastasis compared with paired adjacent normal tissues (Fig. 3C). Furthermore, Kaplan–Meier analysis of primary CRC specimens with long-term follow-up demonstrated that the higher YTHDF3 expression was correlated to poorer overall survival (OS) of CRC patients. The median survival time was about 44 months in CRC patients with lower YTHDF3 expression but only 26 months in CRC patients with higher expression of YTHDF3 (Fig. 3D). However, IHC showed the levels of YTHDF1 and YTHDF2 were not significantly consistently upregulated in primary CRC tissues or liver ­metastasis lesions compared with normal intestinal mucosal tissues (Fig. S5A and S5B). Further multiplexed immunofluorescence staining results showed that the expression of YTHDF3 was mainly in tumor cells, other than T cells, B cells, or macrophages in tumor tissues (Fig. S5C). However, the expression of YTHDF1 and YTHDF2 was both in tumor cells and stromal cells (Fig. S5D and S5E). In addition, we use the GEPIA server to analyze the expression of candidate m^6^A RNA methylation regulatory factors at the mRNA level in 9,736 tumors and 8,587 normal samples from the The Cancer Genome Atlas (TCGA) and the GTEx projects. The results in Fig. S6A showed a substantially upregulated expression of YTHDF3 in tumor tissues compared with normal tissues in different tumor types. Forest plots demonstrated that YTHDF3 increased the hazard of death from CRC (Fig. S6B). Moreover, the expression of ALKBH5, FTO, and METTL3 was down-expressed both in colon adenocarcinoma (COAD) and rectal adenocarcinoma (READ), which were consistent with our transcriptome analysis (Fig. S6C).

In addition, both OA and PA, which promote the accumulation of intracellular LDs, upregulated the expression of YTHDF3 (Fig. 3G and 3I). In contrast, C75, a fatty acid synthase inhibitor, downregulated YTHDF3 expression (Fig. 3J). Oil red O staining indicated the elevated lipids accumulation in HFD feed groups both in subcutaneous xenograft model and splenic injection model of liver metastases in nude mice (Fig. 3F). Western blots analysis hint that the expression of YTHDF3 was positively correlated with lipids deposition both in subcutaneous xenograft model and splenic injection model of liver metastases in nude mice (Fig. 3H and 3K). Taken together, these results identified that lipid accumulation enhances YTHDF3 expression in CRLM, which is correlated with poorer OS of CRC patients.

### YTHDF3 is required for tumor proliferation and invasion

**Figure 4.**
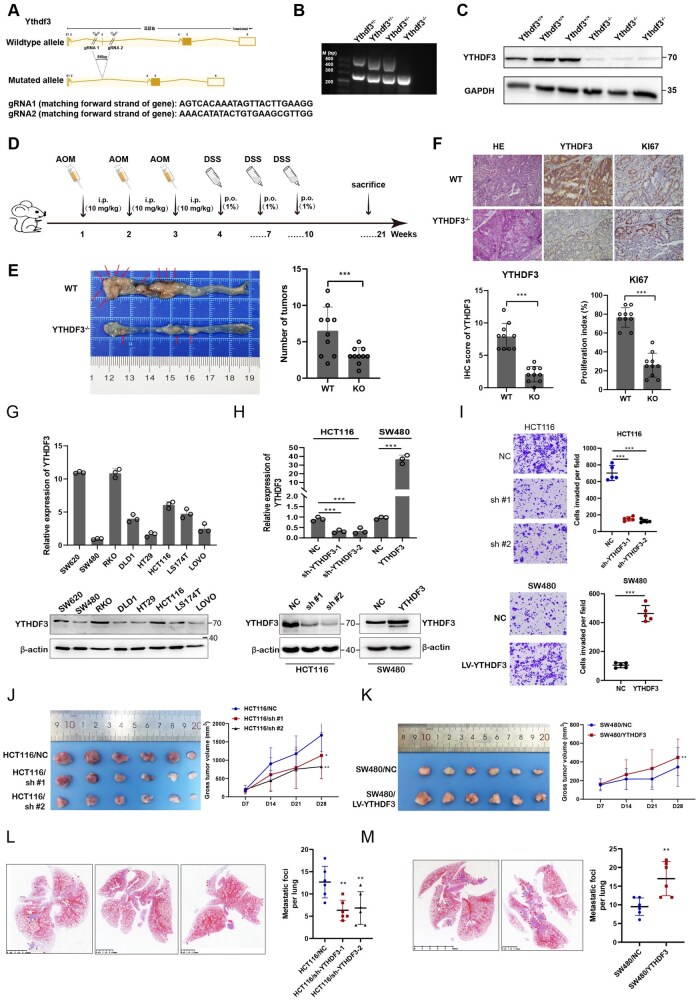
**YTHDF3 promotes CRC progression and metastasis *in vitro* and *in vivo***. (A) gRNA target sequence of Ythdf3 knockout mice. (B and C) PCR and Western blots identified WT or knockout mice. (D) AOM and DSS model of primary colon carcinoma in mice. (E) Ythdf3 knockout inhibits CRC tumor initiation and progression. (F) hematoxylin and eosin (HE) and IHC staining of the indicated proteins. (G) ­qRT-PCR and Western blot analysis showed YTHDF3 expression in various human CRC cells. (H) qRT-PCR and Western blot analysis of YTHDF3 expression in HCT116 cells transfected with YTHDF3-specific shRNA or sh-control, and SW480 cells transfected with YTHDF3-overexpressing lentiviral vector or negative control. All experiments were performed in triplicate, and results are presented as mean ± SD. **P < 0.01, ***P < 0.001. (I) Transwell assays were performed to investigate the changes in invasion abilities of CRC cells, respectively. The mean ± SD is shown for five independent experiments. ***P < 0.001. (J–K) Representative images of tumor growth in xenografted BALB/c nude mice. Each group of mice was ectopically implanted with 2 × 10^6^ indicated cells into the flanks of mice (*n *= 6). Here, cells were transfected with YTHDF3-overexpressing lentiviral vector or YTHDF3-specific shRNA. And the volume of tumors in individual mice was calculated (right panel). Results are presented as mean ± SD. *P < 0.05, **P < 0.01. (L and M) Representative lung tissue images of lung metastasis number and foci are shown by HE staining. And the area of metastases foci in individual mice was calculated using Dmetrix software (right panel). (*n *= 6); **P < 0.01.

As results showed in Fig. 4A–C, we created Ythdf3 knockout mouse model by CRISPR/Cas9-mediated genome engineering. Results of AOM and DSS inducible colorectal carcinoma model showed that the tumor numbers and tumor sizes were decreased in Ythdf3^−/−^ mice (Fig. 4D–F). We constructed human CRC cells stably overexpressing or knockdown of YTHDF3 to further investigate the role of YTHDF3 in CRC progression *in vitro* and *in vivo* (Fig. 4G and 4H). Functional assays showed that stably overexpression of YTHDF3 significantly stimulated cell proliferative and invasion capacity *in vitro* and *in vivo*, whereas knockdown of YTHDF3 reversed the effects (Fig. 4I–M).

Moreover, suppression of YTHDF3 eliminated the enhanced metastatic potential of CRC cells induced by lipid accumulation both *in vitro* and *in vivo* (Fig. S7A–E). The intrasplenic injection liver metastasis models in mice demonstrated that knockdown of YTHDF3 reduced LD deposition and Ki67 expression in the liver metastases induced by the HFD model (Fig. S7F–H).

Furthermore, we established AOM and DSS inducible model of colon carcinoma in Ythdf3^−/−^ mice, and the results showed Ythdf3 knockout attenuates HFD-induced CRC tumor progression (Fig. S8A and S8B). The spatial metabolomics was performed to identify the differentially metabolites in tumor tissues *in situ*. PCA indicated that lipid metabolites in the indicated groups were greatly changed compared with those in wild-type mice with SC (Fig. S8C), as summarized in Table S3. Heatmap plots and K-means clustering analysis of differentially expressed metabolites revealed that Ythdf3 knockout partially mitigated the HFD-induced lipid metabolic alterations (Fig. S8D and S8E). The spatial distribution of differentially expressed lipid metabolites in tumor tissues of indicated groups is shown in Fig. S8F–I.

Collectively, these data suggest that YTHDF3 plays a crucial role in CRLM. Disruption of YTHDF3 attenuates lipid deposition-induced CRC progression and metastasis both *in vitro* and *in vivo*.

### YTHDF3 facilitates m^6^A modification and degradation of PPARα

**Figure 5.**
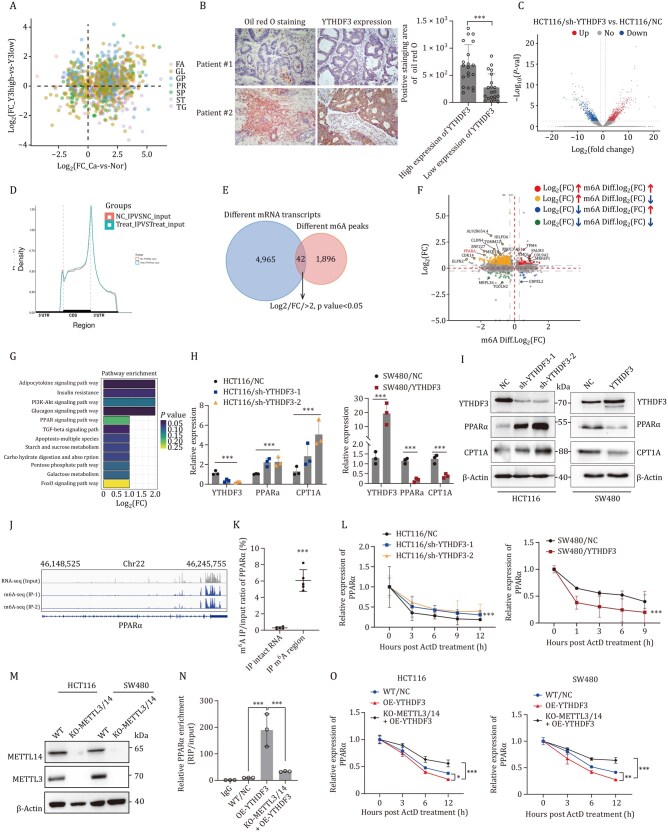
**YTHDF3 facilitates m^6^A modification and degradation of PPARα**. (A) Four-quadrant diagram of differential lipid metabolites in tumor groups with high (FC_Y3high) versus low (FC_Y3low) YTHDF3 expression. *P *< 0.05 and fold change >1.25 or <0.8. (B) Oil red O staining and IHC analysis of YTHDF3 in liver metastasis of CRC patients. (C and D) Transcriptomic (C) and MeRIP sequencing (D) of human CRC cells with inhibited YTHDF3 expression. (E) Venn diagram displaying common differentially expressed genes in RNA-seq and MeRIP-seq (/FC/>2; *P*-value < 0.05). (F) Correlation of differentially expressed transcripts and m^6^A modification transcripts in YTHDF3 knockdown cells (/FC/>2; *P*-value < 0.05). Group 1 (yellow) are upregulated transcripts in RNA-seq but downregulated in MeRIP-seq; Group 2 (red) are upregulated transcripts in both (fold change > 2; *P*-value < 0.05). (G) KEGG pathway enrichment of differentially expressed genes in both transcription-seq and MeRIP-seq (/FC/> 2; *P*-value < 0.05). (H) qRT-PCR detection of indicated gene expression. Experiments performed in triplicate, results presented as mean ± SD. ***P < 0.001. (I) Western blots show gene expression in CRC cells with YTHDF3 overexpression or silencing. (J) Integrative Genomics Viewer (IGV) analysis shows m^6^A peaks among PPARα mRNA. (K) m^6^A levels of PPARα quantified by MeRIP qRT-PCR in HCT116 cells. Mean ± SD shown for five independent experiments. ***P < 0.001. (L) qRT-PCR of PPARα in actinomycin D-treated CRC cells. Actinomycin D (100 nM) inhibits transcription of the indicated gene. Mean ± SD shown for five independent experiments. ***P < 0.001. (M) METTL3/14-DKO CRC cells were generated using CRISPR-Cas9. (N) MeRIP-qPCR analysis in indicated cells. (O) PPARα mRNA stabilization was detected in the indicated cells.

Through integrative analysis of transcriptome and lipidomics, we identified that lipids metabolites were elevated in liver metastases which was consistent with that in the group of tumor high expression of YTHDF3 (Fig. 5A). Oil red O and IHC analysis showed YTHDF3 expression was positively correlated with lipids deposition in CRLM (Fig. 5B). Furthermore, we transfected HCT116 cells with YTHDF3 siRNAs and then performed MeRIP sequencing combined with transcriptome analysis to verify the target gene of YTHDF3 (Fig. 5C). The MeRIP sequencing showed that the 3′-untranslated region (3′-UTR) contained the highest percentage of mRNA m^6^A modification (Fig. 5D). Venn diagram showed differentially expressed transcripts both in m^6^A modification and transcription (Fig. 5E; Table S4). Most importantly, transcriptome analysis displayed that the majority of the m^6^A-related mRNA transcripts were upregulated in a group of cells transfected with YTHDF3-specific siRNAs (Fig. 5F). Furthermore, RNA lifetime profiling analysis showed YTHDF3 overexpression shortened the stability of m^6^A-containing mRNAs, whereas knockdown of YTHDF3 yielded the opposite results (Fig. S9). These results implicated that YTHDF3 was related to mRNA degradation in CRC cells. Kyoto Encyclopedia of Genes and Genomes (KEGG) pathway analysis enriched in metabolic processes (Fig. 5G). Further quantitative reverse-transcription PCR (qRT-PCR) and Western blot analyses demonstrated that PPARα, the key regulator of FAO, was the only consistently dysregulated gene among these differentially expressed genes. YTHDF3 knockdown elevated the mRNA and protein levels of the PPARα-CPT1A signaling pathway, whereas YTHDF3 overexpression suppressed the expression of these indicated genes (Fig. 5H and 5I).

Further integrative genomics viewer analysis of MeRIP-seq identified that m^6^A peaks among PPARα transcripts were in the 3′-UTR region, which contains the YTHDF3-binding motif UGGACU (Fig. 5J). MeRIP and qRT-PCR assays showed that the binding capacity of YTHDF3 and PPARα was increased in the m^6^A modification region (Fig. 5K). RNA lifetime profiling results showed exogenous expression of YTHDF3 led to shorten lifetime of PPARα compared with the negative control. Whereas, inhibited YTHDF3 expression led to prolonged lifetime of PPARα transcript (Fig. 5L). To demonstrate that the accelerated decay of PPARα mRNA observed upon YTHDF3 overexpression is m^6^A-dependent, we have generated METTL3/14 double-knockout (DKO) CRC cells (Fig. 5M). Further MeRIP-qPCR demonstrated that m^6^A enrichment on PPARα is abolished in METTL3/14-DKO cells, confirming the loss of the m^6^A mark (Fig. 5N). In METTL3/14-DKO cells, PPARα mRNA is no longer sensitive to YTHDF3, demonstrating that the destabilizing effect of YTHDF3 is strictly m^6^A-dependent (Fig. 5O). Collectively, these results indicated that YTHDF3 facilitates m^6^A modification and degradation of PPARα.

Functional assays showed that overexpression of PPARα significantly suppressed CRC cell proliferation and invasion *in vitro* and *in vivo* (Fig. S10A–C). Furthermore, PPARα overexpression abrogated lipid accumulation-induced promotion of tumor metastasis *in vitro* and *in vivo* (Fig. S10D–F). Further function assays showed PPARα reversed YTHDF3-mediated promotion and invasion of CRC *in vitro* and *in vivo* (Fig. S11). Fenofibrate, a PPARα agonist, was tested in rescue assays. Low-level PPARα activation halved the invasion of wild-type (WT) CRC cells, and high-level activation was required to curb their growth. In contrast, neither dose significantly reversed the heightened proliferation or invasion of YTHDF3-overexpressing cells (Fig. S12A–F). Furthermore, we re-expressed YTHDF3 or knocked out PPARα in YTHDF3-KO CRC cells. Only the re-expression of YTHDF3 fully restored the proliferation and invasion capacities; the knockout of PPARα alone in the YTHDF3-KO background did not fully restore these capacities, indicating that additional targets of YTHDF3 (beyond PPARα) do contribute. However, PPARα-KO abolishes nearly 70% of the anti-metastatic effect of YTHDF3 knockdown, confirming that PPARα is the non-redundant mediator of YTHDF3-driven metastasis (Fig. S12G–J).

Collectively, these results indicated that YTHDF3 facilitates m^6^A modification and degradation of PPARα. Targeting the YTHDF3-PPARα axis could provide potential interventions to prevent CRLM.

### Lipid accumulation attenuates PPARα-mediated β-hydroxybutyrylation, which is crucial for CRC progression

**Figure 6.**
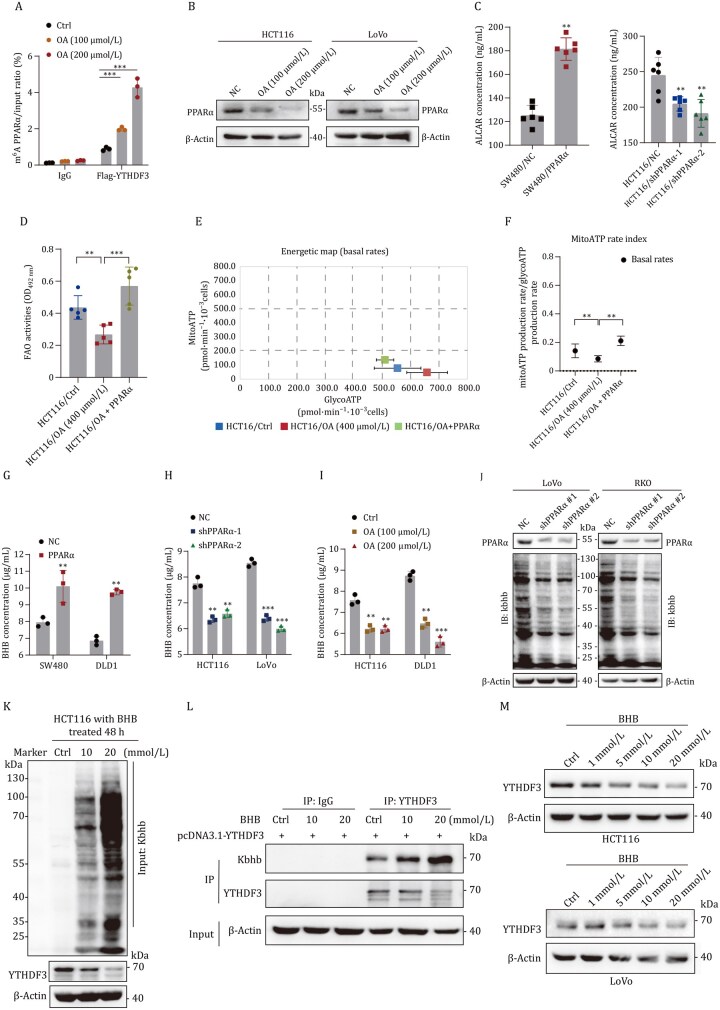
**Lipid accumulation attenuates PPARα-mediated β-hydroxybutyrylation**. (A and B) MeRIP qRT-PCR and Western blot assay showed OA increased m^6^A modification of PPARα and declined PPARα expression. (C) ELISA showed acetyl-L-carnitine production in the indicated groups. (D) Fatty acid oxidation assay showed PPARα reversed OA-induced FAO activity suppression. (E and F) Seahorse assay revealed the mitoATP ratio and glycoATP ratio in the indicated groups. (G–I) ELISA showed BHB production in the indicated groups. (J and K) Kbhb modification in the indicated groups. (L and M) BHB increased Kbhb-modified YTHDF3 (L) and decreased the total protein of YTHDF3 (M).

MeRIP qRT-PCR and Western blot assay showed OA excessively increased m^6^A modification of PPARα and declined PPARα expression (Fig. 6A and 6B). The FAO assay and Seahorse assay revealed that overexpression of PPARα reversed the OA-induced glycolytic ATP ratio, while concurrently increasing the mitoATP ratio (Fig. 6D–F). ELISA showed that overexpression of PPARα significantly increased metabolites of FAO, such as acetyl-L-carnitine (ALCAR) and BHB (Fig. 6C, 6G, and 6H). Whereas, increased OA concentration declined BHB production in CRC cells (Fig. 6I).

BHB is employed in the regulation of numerous proteins, conferring an additional layer of regulation upon diverse cellular and metabolic pathways. However, the impact and mechanism of BHB and Kbhb modification in tumor metastasis remain largely unknown. Notably, we found the Kbhb modification was suppressed in CRC cells knockdown of PPARα (Fig. 6J). To assess the extent of protein Kbhb modification in vitro, cultured CRC cells were treated with Na-β-OHB for 48 h. CRC cells displayed concentration-dependent increases in protein Kbhb in response to treatment (Fig. 6K). To characterize the specific proteins and sites of Kbhb, a polyclonal pan-β-hydroxybutyryllysine antibody was used for immunoprecipitation analysis and followed by liquid chromatography-tandem mass spectrometry (LC-MS/MS). We identified 1,053 sites of Kbhb across 916 proteins in BHB-treated CRC cells. Notably, the analysis showed the DTQEVPLEK site of the YTHDF3 protein was enrichment, indicating Kbhb modification of YTHDF3 (Table S6). Further Western blot showed that BHB increased Kbhb-modified YTHDF3 compared to control conditions, while the total protein level of YTHDF3 declined (Fig. 6L). Further analysis revealed that the total protein of YTHDF3 had declined in a concentration-dependent manner in Na-β-OHB (Fig. 6M).

**Figure 7.**
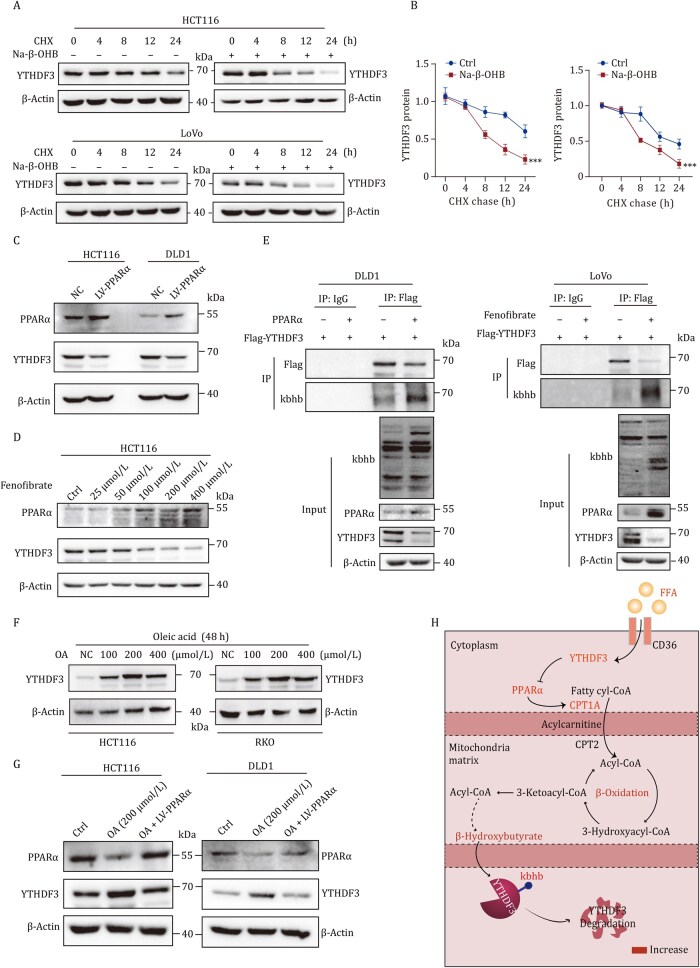
**Lipid deposition attenuates Kbhb-modified YTHDF3**. (A and B) CHX chase assay showed that BHB significantly increased degradation of the YTHDF3 protein. (C and D) Western blot showed PPARα and YTHDF3 protein levels of the indicated groups. (E) CO-IP assay showed Kbhb-modified YTHDF3 in the indicated groups. (F and G) Western blot showed PPARα and YTHDF3 protein levels of the indicated groups. (H) Schematic diagram shows that PPARα facilitates BHB production and Kbhb modification and degradation of YTHDF3.

Despite the biological importance of Kbhb, little is known about its function in tumor progression. Thus, we sought to further investigate the regulatory effect of Kbhb modification on YTHDF3. Cycloheximide (CHX) chase assay showed that BHB significantly increased degradation of YTHDF3 protein (Fig. 7A and 7B). Western blot results showed that overexpression of PPARα or treated with fenofibrate, an agonist for PPARα, significantly declined YTHDF3 expression in CRC cells (Fig. 7C and 7D). Co-immunoprecipitation (CO-IP) assay showed that overexpression of PPARα or treated with fenofibrate increased Kbhb-modified YTHDF3 and decreased the expression of YTHDF3 (Fig. 7E). As we identified that PPARα overexpression reversed lipid deposition-mediated elevation of YTHDF3 protein (Fig. 7F and 7G). As shown in Fig. S10G, the Kbhb modification was decreased in primary CRC tissues and paired liver metastases compared with paired normal tissues. Further liver metastasis model of CRC cells in mice identified that BHB intragastric administration decreased liver metastases of CRC cells *in vivo* (Fig. S10H). Collectively, these data reveal a feedback loop between PPARα and YTHDF3, through which PPARα promotes Kbhb-modified YTHDF3 to degradation, which is attenuated by lipid accumulation in CRLM (Fig. 7H).

### Lipid deposition induces liquid–liquid phase separation of YTHDF3

**Figure 8.**
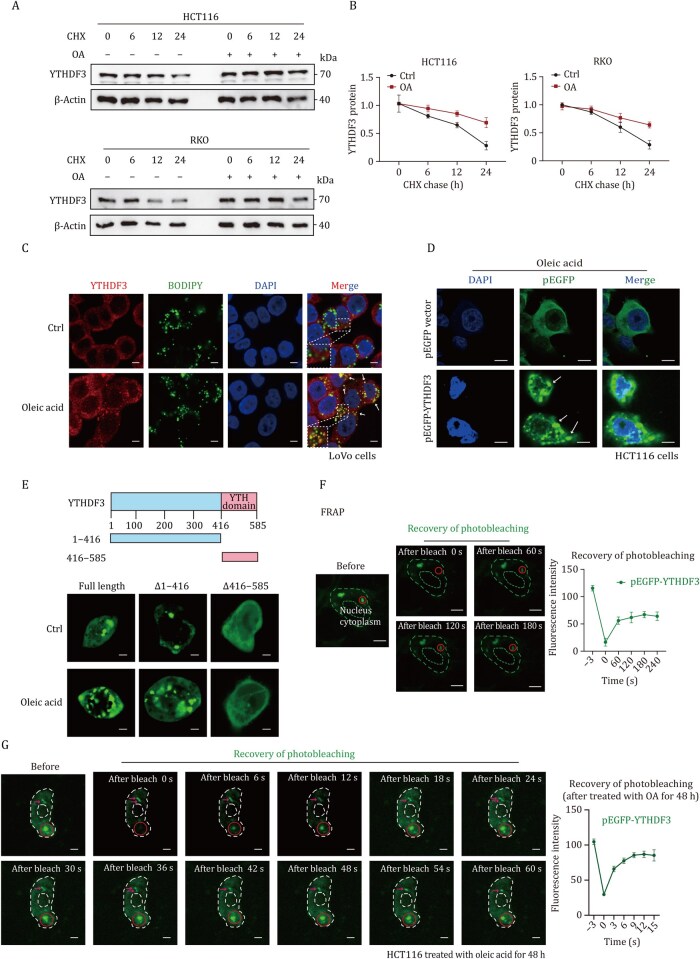
**Lipid accumulation triggers LLPS of YTHDF3**. (A and B) CHX chase assays showed that the half-life of the YTHDF3 protein was remarkably increased when CRC cells were treated with OA. (C) Immunofluorescence showed OA enhanced co-localization of YTHDF3 protein and LDs. The ruler represents a scale of 5 µm. (D) Immunofluorescence showed YTHDF3-EGFP formed puncta in the cytoplasm of CRC cells when treated with OA. The ruler represents a scale of 5 µm. (E) Mapping the YTHDF3 domains required for LLPS. The ruler represents a scale of 5 µm. (F and G) FRAP assay of YTHDF3 puncta in CRC cells with OA treatment. Fluorescence intensity curves are shown (right panel). The ruler represents a scale of 5 µm. Data are presented as mean ± SD.

LLPS has been known for its specific functions in biological processes. The YTH domain family proteins undergo LLPS, which is subject to compartment-specific regulation, regulating protein stability and translation. However, whether lipids accelerate LLPS progression remains unknown. We have identified that lipid deposition elevated YTHDF3 expression; furthermore, CHX chase assays showed that the half-life of YTHDF3 protein was remarkably increased when CRC cells were treated with OA (Fig. 8A and 8B). Intriguingly, we found lipid deposition significantly increased phase separation and condensate formation of YTHDF3 and excessively increased co-localization of YTHDF3 protein and LDs (Fig. 8C and 8D). To visualize the co-localization of YTHDF3 and LDs, we employed an ultra-high-resolution microscope to track the dynamic interactions between EGFP-labeled YTHDF3 protein and LDs in CRC cells. The results showed that the YTHDF3 protein rapidly underwent fusion and fission events in response to the movement of LDs (Fig. S13A; Video S1). Immunofluorescence assays revealed that lipid deposition enhanced the co-localization of YTHDF3 with PLIN3, a key protein of the LD surface, in CRC cells (Fig. S13B). Subsequent co-immunoprecipitation experiments validated the interaction between YTHDF3 and PLIN3 proteins in CRC cells treated with OA (Fig. S13C). These results uncovered that lipid deposition significantly enhances the co-localization of YTHDF3 at LDs, thereby promoting the LLPS formation of YTHDF3.

**Figure 9.**
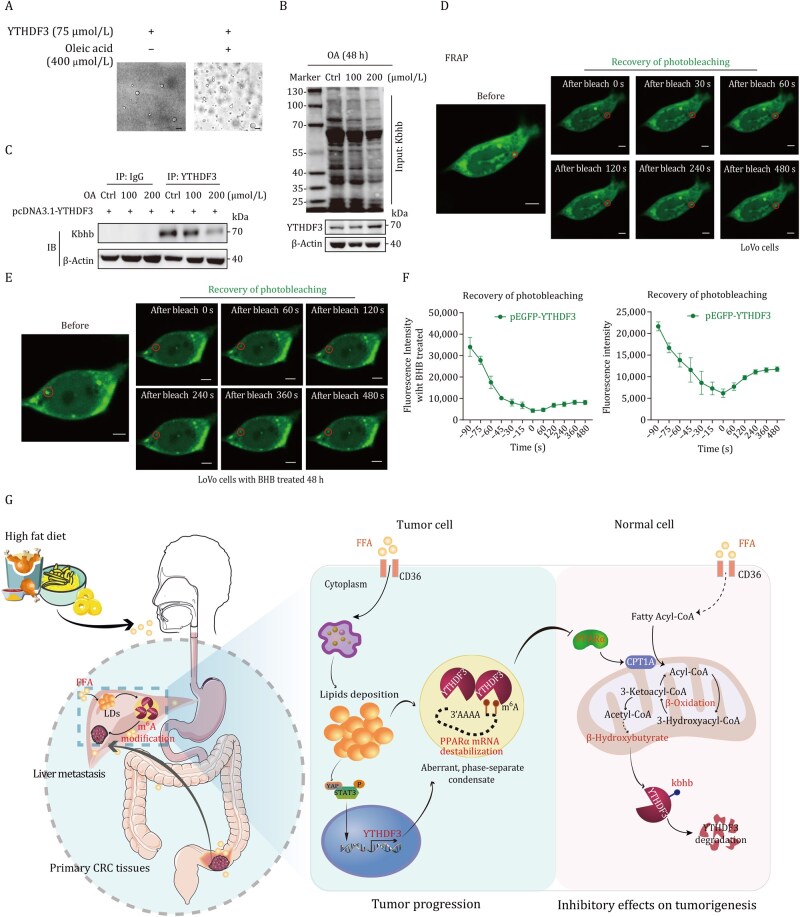
**BHB inhibits LLPS of YTHDF3**. (A) Bright-field microscopy images showed OA triggers YTHDF3 protein LLPS *in vitro*. (B and C) OA suppressed Kbhb modification (B) and Kbb-modified YTHDF3 (C) in CRC cells. (D–F) FRAP assay of YTHDF3 showed that the photobleaching region of YTHDF3 puncta was recovered more slowly in BHB-treated CRC cells. Fluorescence intensity curves are shown (F). The ruler represents a scale of 5 µm. Data are presented as mean ± SD. (G) Schematic illustration of lipid deposition fostering CRC progression and metastasis through YTHDF3-mediated m^6^A modification of PPARα and inhibition of β-hydroxybutyrylation.

We purified recombinant YTHDF3 protein and found that OA promotes phase separation condensate formation of YTHDF3 in vitro (Fig. 9A). To map the domains of YTHDF3 required for LLPS, we tested truncated forms (YTH domain and N-terminal mutation with non-m^6^A recognition region) of YTHDF3 protein, and found that N-terminal mutation (1-416 aa) was required for LLPS. Lipid deposition significantly enhanced LLPS of N-terminal mutation of YTHDF3 (Fig. 8E). Furthermore, we performed fluorescence recovery after photobleaching (FRAP) assay of YTHDF3 puncta in CRC cells with OA-treated or not. The photobleaching region of YTHDF3 droplet showed rapid recovery of fluorescence in OA-treated CRC cells, and the half-life of recovery is 2.857 s, whereas the recovery time was longer than 4 min in cells not treated with OA (Fig. 8F and 8G). However, further co-immunoprecipitation and Western blot assay showed that lipid deposition attenuated total protein Kbhb modification and Kbhb-modified YTHDF3 (Fig. 9B and 9C). FRAP assay showed that the photobleaching region of YTHDF3 puncta recovered more slowly in BHB-treated CRC cells compared with the negative control (Fig. 9D–F).

These findings initially establish a link between lipid deposition and LLPS within a post-translational regulatory mechanism. This elucidates that lipid deposition promotes YTHDF3-mediated m^6^A modification, which attenuates β-hydroxybutyrylation to facilitate liver metastasis of CRC.

### Lipid deposition enhances the interaction between STAT3 and YAP and facilitates the expression of YTHDF3

Previous studies have reported that obesity can induce the activation of the STAT3 (signal transducer and activator of transcription 3) signaling pathway. The JAK/STAT3 pathway is observed to be activated in several pathological processes, including NAFLD and cancer. We examined and verified that the expression of pSTAT3^Tyr705^ was stimulated in OA-treated CRC cells in a dose-dependent manner (Fig. S14A). However, NF-κB, another key transcription factor involved in inflammation, was not altered. More importantly, the knockdown of STAT3 significantly reduced the expression of YTHDF3 in CRC cells treated with OA (Fig. S14B and S14C). However, in the absence of OA, overexpression of STAT3 did not alter the mRNA and protein expression of YTHDF3. More intriguingly, we discovered that the expression of Yes-associated protein (YAP) was upregulated in a dose-dependent manner in CRC cells treated with OA (Fig. S14D). Additionally, co-immunoprecipitation analysis revealed that the interaction between YAP and STAT3 was significantly enhanced in CRC cells upon OA stimulation (Fig. S14E). Furthermore, the expression of YTHDF3 was significantly reduced when co-transfected with specific siRNAs targeting YAP and STAT3 (Fig. S14F). Immunofluorescence analysis revealed a significant correlation between STAT3 and nuclear YAP in CRC cells upon OA stimulation (Fig. S14G). The JASPAR database predicted that the region from −500 to +100 bp upstream of the YTHDF3 transcriptional start site contains binding sites for STAT3 and YAP. A subsequent ChIP-qPCR assay indicated that YTHDF3 is a novel target of both STAT3 and YAP (Fig. S14H).

We further utilized the HDOCK server to predict and generate the structure of the STAT3-YAP interacting model and the YTHDF3 promoter complexes. Initially, we employed AlphaFold to ascertain the structure of the STAT3 and YAP protein interaction model (Fig. S15A), and the 3D structure of the YTHDF3 promoter was forecasted using 3dRNA/DNA software (Fig. S15B). As depicted in Fig. S15C, the structure of the STAT3-YAP interacting complex is depicted in orange cartoon, while the YTHDF3 transcriptional start site is shown in cyan. The binding sites between the protein and promoter are denoted by a dashed box. The structural figures were produced using HDOCK.

Additionally, in our transcriptomic analysis, we observed that the JAK/STAT pathway was significantly upregulated in CRC tissues compared to normal intestinal mucosa (Fig. S16A). Correlation analysis revealed a positive correlation between STAT3 and YTHDF3 in CRC tissues within the TCGA database (*r*^2^ = 0.37; *P *< 0.001) (Fig. S16B). Conversely, the PPAR-CPT1 signaling pathway was downregulated in CRC tissues (Fig. S16C). A positive correlation was also found between PPARα and CPT1A in CRC tissues within the TCGA database (*r*^2^ = 0.54; *P *< 0.001) (Fig. S16D). Furthermore, Kaplan–Meier analysis indicated that higher STAT3 expression was associated with poor OS in CRC patients, while increased expression of PPARα and CPT1A correlated with better prognosis for these patients (Fig. S16E–G). These findings suggest that lipid accumulation enhances the STAT3–YAP interaction and promotes YTHDF3 expression.

Collectively, we discovered that lipid deposition simultaneously enhances YTHDF3 transcription via STAT3–YAP binding and inhibits its degradation by suppressing Kbhb modification, thereby collectively promoting YTHDF3 accumulation in lipid-rich metastases. Targeting the YTHDF3-PPARα axis could offer potential interventions to prevent liver metastasis of CRC (Fig. 9G).

## Discussion

The liver is an important target organ for metastasis in many types of cancer, including CRC (Hess *et al.*, 2006). Recent evidence has demonstrated aberrantly regulated lipid metabolism as a high risk for liver metastasis; the underlying molecular mechanism remains underway (Brouquet and Nordlinger, 2013; Hamady *et al.*, 2013; Lv and Zhang, 2020; Shen *et al.*, 2010; Wang *et al.*, 2023). Key enzymes involved in the uptake, synthesis, and lipolysis of lipids play crucial roles in tumors (Zhou *et al.*, 2022). Previous studies showed that HFD is not only associated with the progression of the primary tumor, but is also very important for tumor recurrence and metastasis (Martin-Perez *et al.*, 2022). However, the underlying mechanisms of liver metastasis are still a pivotal perplex to be fully elucidated.

Herein, we found that lipid metabolic reprogramming is a common feature for liver metastases of CRC. In clinical samples including PDOs, lipid metabolites were accumulated in CRLM. Importantly, lipid deposition promotes liver metastasis of CRC *in vitro* and *in vivo*. Our study also showed that restraining lipid deposition, such as TRF, greatly decreased liver metastasis of CRC in mice. Mechanistically, HFD continuously supplies exogenous lipids, which overwhelm the PPARα-mediated β-oxidation pathway and sustain YTHDF3-driven m^6^A modification of PPARα, thereby promoting LLPS and metastasis. In contrast, TRF establishes circadian metabolic rhythms that enhance FAO and elevate intratumoral levels of BHB. The increased BHB promotes Kbhb of YTHDF3, thereby accelerating its proteasomal degradation and subsequently suppressing LLPS and tumor progression. Consequently, TRF mitigates the pro-metastatic effects of HFD by restoring the regulatory PPARα-BHB-YTHDF3 feedback loop. Thus, the metabolic interventions aiming to reduce lipid deposition will provide prospective therapeutic opportunities to prevent liver metastasis of CRC. In CRC patients, high YTHDF3 expression is positively correlated with elevated serum levels of lipid transport proteins (APOB, APOA1; *P *< 0.01), suggesting a clinically relevant link between YTHDF3-driven lipid metabolism and tumor progression. It is important to emphasize that, although murine data firmly establish HFD as an accelerator of YTHDF3-mediated CRLM, human evidence linking dietary fat intake to metastatic progression is strictly correlative. Definitive proof awaits prospective, intervention-based trials in high-risk CRC cohorts specifically designed to track dietary fat modification and its mechanistic impact.

Furthermore, our study revealed that the YTHDF3-mediated m^6^A modification of PPARα is a crucial mechanism in the development of CRLM. PPARα is a significant transcription factor that regulates genes involved in FAO (Bocher *et al.*, 2002; Fujinuma *et al.*, 2023; Walczak and Tontonoz, 2002). PPARα agonists have been demonstrated to play a pivotal role in the prevention and treatment of NAFLD by modulating lipid metabolism and reducing liver fat accumulation (Bocher *et al.*, 2002). However, the role of PPARα in liver metastasis was previously unclear. In this study, we discovered that lipid deposition significantly promotes the YTHDF3-mediated m^6^A modification and degradation of PPARα, which is essential for CRLM. Several recent reports, including our own, describe YTHDF3 as an m^6^A reader that stabilizes oncogenic transcripts such as MYC, EGFR, and PFKL, thereby accelerating proliferation and angiogenesis (Chang *et al.*, 2020; Chen *et al.*, 2024; Ni *et al.*, 2019; Zhou *et al.*, 2022). Independent studies have shown that PPARα agonists (such as fenofibrate) inhibit the growth of CRC cells in vitro and decrease polyp burden in mice (Luo *et al.*, 2019; Saidi *et al.*, 2006). While earlier research focused on PPARα-mediated transcriptional repression of lipogenic enzymes, our findings uncover an m^6^A-dependent post-transcriptional mechanism in which PPARα is destabilized by YTHDF3, creating a self-amplifying oncogenic loop. Moreover, this study indicates that PPARα is uniquely positioned at the intersection of lipid metabolism and invasion: it is the only m^6^A-marked ­transcript whose loss simultaneously (i) suppresses FAO, (ii) decreases BHB production, and (iii) stabilizes YTHDF3 through reduced Kbhb. Functionally, the reactivation of PPARα reversed the proliferation and invasion driven by YTHDF3 both *in vitro* and *in vivo*; fenofibrate only partially rescued YTHDF3-overexpressing cells, suggesting additional targets. However, the knockout of PPARα alone restored the pro-metastatic phenotype lost upon deletion of YTHDF3, establishing PPARα as the non-redundant downstream effector. Targeting the YTHDF3–PPARα axis is a functionally significant, though not the sole, mechanism underlying YTHDF3-mediated CRC progression.

Moreover, we identified a self-reinforcing feedback loop that couples lipid accumulation to the upregulation of YTHDF3. At the post-translational level, excess lipids suppress PPARα-mediated Kbhb modification of YTHDF3, thereby extending its half-life and promoting its LLPS, leading to the formation of a specialized compartment for m^6^A-RNA processing. At the transcriptional level, lipid overload enhances the interaction between STAT3 and YAP, which in turn upregulates YTHDF3 expression. These two regulatory mechanisms synergize to drive YTHDF3 accumulation in lipid-rich metastatic lesions. Our study firstly establishes links between lipid deposition and LLPS through an m^6^A-dependent regulatory mechanism, highlighting its critical role in liver metastasis.

Intriguingly, we uncovered that lipid deposition markedly enhanced the co-localization of YTHDF3 with LDs, thereby facilitating the LLPS of YTHDF3. In membrane model systems, the lipid-lipid interaction is essential for LLPS formation, which may influence LLPS formation by modifying lipid fusion and fission events and viscosity (Schanen and Petty, 2023; Sych *et al.*, 2021). For instance, lipid phase segregation in B-cell receptors can recruit Lyn kinases to lipid-ordered regions while repelling phosphatases such as CD45, thus amplifying signaling (Stone *et al.*, 2017). LLPS is an emerging concept in cellular biology that plays significant roles in tumor biology, which has been linked to several hallmarks of cancer (Mehta and Zhang, 2022; Zhang *et al.*, 2020). However, whether lipid deposition promotes LLPS contributes to the liver metastasis of tumors remains unclear. Our study revealed a previously unrecognized yet critical mechanism by which lipid deposition facilitates the LLPS of YTHDF3 through the co-localization of YTHDF3 protein with LDs. At the protein level, lipid deposition forms a special niche for RNA m^6^A modification. This leads to increased stability of YTHDF3 to promote CRC progression and liver metastasis.

Collectively, this study uncovered that lipid deposition promotes LLPS-mediated m^6^A modification and attenuates the β-hydroxybutyrylation of YTHDF3 in liver metastasis, providing novel strategies for the treatment of CRLM.

## Materials and Methods

### Data availability statement

The transcript sequencing data of CRC tissues from clinical patients and the MeRIP sequencing data discussed in this paper have been deposited in NCBI’s Gene Expression Omnibus and accessible through GEO Series accession number GSE129716 and GSE221608. The data will become public when this article is published online. Other data in this study are available from the corresponding author on reasonable request.

### Tissue samples and immunohistochemistry (IHC) staining

All matched tissue samples were collected simultaneously from the same patient during a single surgical operation. Specifically, normal intestinal mucosa (Nor) was obtained from histologically confirmed tumor-free margins (>2 cm from the primary tumor lesion). Primary colorectal cancertissue (Ca) was derived from the primary tumor site, while liver metastasis (LM) samples were obtained from synchronous metastatic lesions in the liver. None of the patients received neoadjuvant chemotherapy or radiotherapy prior to surgery. Detailed patient information, including surgical site and pathological staging, is summarized in Table S5. The median time interval between the resection of the primary tumor and the corresponding liver metastasis was less than two hours. Formalin-fixed paraffin-embedded (FFPE) CRC tissues and adjacent normal tissues were collected from the Department of Pathology at Sun Yat-sen Memorial Hospital, Sun Yat-sen University (Guangzhou, China). The study was approved by the Ethics Committees of Sun Yat-sen University with Approval No. SYSKY-2023-012-01. IHC was performed as we previously described. And IHC staining scores were evaluated in a blinded fashion. The scoring system from 0 to 12 combined the intensity and percentage (signal: “0,” no staining; “1,” weak staining; “2,” intermediate staining; and “3,” strong staining; percentage: “0,” 0%; “1,” 1%–25%; “2,” 26%–50%; “3,” 51%–75%; “4,” >75%), which were used as we described previously. The median value of total staining scores was identified as the optimal cut-off value.

### Tumor dataset acquisition and process

The datasets and corresponding clinical data presented in our study were downloaded from the Cancer Genome Atlas (TCGA) and Genotype-Tissue Expression (GTEx) data portal. Tumor RNA-seq data can be downloaded from the Genomic Data Commons (GDC) data portal website. We used R software to calculate the difference expression between normal and tumor samples in each tumor. Univariate cox regression analysis and forest plots through the “forestplot” R package was used to display the P value, HR and 95% CI of each variable. R software (version 4.0.3) was used for statistical analysis. If not otherwise stated, unpaired Wilcoxon rank sum and signed rank tests were used for significance analysis, the asterisk represents the degree of significance (*p), and P value <0.05 is considered statistically significant.

### Animal models

The animal studies were conducted in accordance with the guidelines approved by the Institutional Animal Care and Use Committee of Sun Yat-sen University with Approval No.SYSU-IACUC-2022-B0790. C57BL/6 mice were purchased from Cyagen Biosciences Inc., and Ythdf3 knockout (Ythdf3−/−) mice, in which exon 3 of Ythdf3 gene was deleted by a CRISPR/ Cas9 System, were established in our lab. All mice were maintained under SPF conditions of Sun Yat-sen University Animal Center. The azoxymethane (AOM) and dextran sulfate sodium (DSS) model of colon carcinoma has been described previously. 6–8-week-old mice were injected with 10mg/kg body weight AOM once a week for three times. And mice were typically subjected to three cycles of weekly 1% DSS exposures, each followed by a 2-week rest period. The animals were sacrificed 20 weeks to measure the number and incidence of tumors. For xenografts tumor model, 2 X 106 cells were subcutaneously injected into nude mice (BALB/c, SPF grade, 6–8 weeks old, n = 6 per group). And tumor sizes were monitored every 3 days for 3 weeks. For in vivo pulmonary metastasis, 1 x 10^6 CRC cells were injected via the tail vein, leading to lung metastasis within 4–6 weeks (with a success rate of ≥ 99%). The intrasplenic injection liver metastasis model involves a caudal spleen injection followed by a hemi-splenectomy, resulting in liver metastasis within 4–6 weeks (with a success rate of ≥ 99%). These models are characterized by a high success rate (with over 99% of mice developing metastases), a short experimental timeline (4–6 weeks), and reproducibility and standardization, making them ideal for studying metastatic colonization and therapeutic interventions. For constructing a time-restricted feeding (TRF) model in mice, we choose a duration of 8 hours (from 7 PM to 3 AM) as feeding window to mimic the benefits of intermittent fasting. Then animals were sacrificed 45 days and the ratio of tumor metastasis loci was calculated.

### Construction of patient-derived organoids (PDOs) models

The study received approval from the Human Research Ethics Committees of Sun Yat-sen University and adheres to all relevant ethical regulations for human research participants. Informed consent was obtained from all subjects. To construct PDOs, we collected tumor specimens from patients with colorectal cancer with separate liver metastases and cultured tumor tissues in a specialized medium that promotes the growth of organoid structures (Precedo, China, Cat.PRS-ICM-3D). The sample should be collected in a sterile container and transported to the laboratory as soon as possible. The tumor tissue is dissociated into single cells, which are embedded in a matrix and plated in a 3D culture system (Precedo, China, Cat. PRS-LM5). The cells are cultured in a humidified incubator at 37°C with 5% CO2. Over time, the cells self-organize and form three-dimensional structures that resemble the original tissue or organ. The organoids can be passaged and expanded for further experiments. HE and IHC staining of PDOs were used to identify of CRC tissues. The data will become public when this article is published online. The study received approval from the Human Research Ethics Committees of Sun Yat-sen University and adheres to all relevant ethical regulations for human research participants. Informed consent was obtained from all subjects.

### Widely-targeted lipidomic analysis and ELISA assay

For Widely-targeted lipidomic analysis, sample was thawed on ice and taken 20 mg of one sample and homogenized it with 1mL mixture (include methanol, MTBE and internal standard mixture) and steel ball. Take out the steel ball and whirl the mixture for 15min. Add 200 uL of water and whirl the mixture for 1 min, and then centrifuge it with 12,000 rpm at 4 °C for 10 min. Extract 300 uL supernatant and concentrate it. Dissolve powder with 200 uL mobile phase B, then stored in -80 °C. Finally take the dissolving solution into the sample bottle for LC-MS/MS analysis. Significantly regulated metabolites between groups were determined by VIP≥ 1 and absolute Log2FC (fold change) ≥1. VIP values were extracted from OPLS-DA result, which also contain score plots and permutation plots, was generated using R package MetaboAnalystR. The data was log transform (log2) and mean centering before OPLS-DA. In order to avoid overfitting, a permutation test (200 permutations) was performed.

For ELISA assay, after cutting specimens, weigh 1 g of tissue and homogenize the specimens adequately by homogenizer. Centrifuge for about 20 minutes (2000 rpm) and carefully collect the supernatant. Pack one part to be tested and the rest to be frozen for reserve. Then add standard sample diluent and incubate for 30minutes at 37°C. Add HRP-Conjugate reagent and incubate for 30 minutes at 37°C. Then add chromogen solution A and B and incubate for 10 minutes at 37°C. Finally, add stop solution and read absorbance at 450nm within 15minutes.

### Fatty acid oxidation (FAO) and Seahorse Real-Time ATP Rate assay

Fatty acid oxidation (FAO) activity was measured using the FAO Assay Kit (Assay Genie, Dublin, Ireland; Cat# BR00001) following the manufacturer’s instructions. Briefly, cells were lysed on ice for 5 minutes, then centrifuged at 14,000 rpm for 5 minutes, and the supernatant was collected. Protein concentration was determined using the BCA assay. Equal amounts of protein (typically 10–20 µg) were added to a 96-well plate in duplicate. Control solution (without substrate) and reaction solution (containing 20× FAO substrate, octanoyl-CoA) were added to respective wells. After incubation at 37°C for 30–120 minutes, the reaction was stopped by adding 3% acetic acid. The absorbance was measured at 492 nm using a microplate reader. FAO activity was calculated by subtracting the control well absorbance from the reaction well absorbance. This assay detects NADH generation coupled with the reduction of INT (a tetrazolium salt), forming a red formazan product proportional to FAO activity. Octanoyl-CoA is used as a medium-chain fatty acid substrate to ensure solubility and mitochondrial enzyme accessibility.

The Seahorse Real-Time ATP Rate Assay is performed according with the manufacturer’s instructions (Agilent, California, USA). Firstly, prepare assay media and Seahorse XF cell culture microplate for assay. Then, metabolic modulators (oligomycin and a mix of rotenone and antimycin A) were serially injected, allow the calculation of the mitochondrial and glycolytic ATP production rates. After running assay, using the Agilent Seahorse XF Real-Time ATP Rate Assay Report Generator to calculates the XF Real-Time ATP Rate Assay Parameters (mitoATP Production Rate, glycoATP Production Rate, total ATP Production Rate, XF ATP Rate Index, % glycolysis, and % OXPHOS). The quantification of the “glycolytic ATP ratio” and “mitoATP ratio” was conducted using the Agilent Seahorse XF Real-Time ATP Rate Assay Kit (Cat# 103592-100), following the manufacturer’s protocol. Baseline measurements of oxygen consumption rate (OCR) and extracellular acidification rate (ECAR) were taken before the injection of oligomycin (1 µM) to inhibit mitochondrial ATP production. Subsequently, rotenone and antimycin A (0.5 µM each) were administered to halt mitochondrial respiration. The values were automatically computed using Agilent Seahorse Wave Desktop v2.6 software and presented as the mean ± standard deviation (SD) from four separate experiments, with five wells per group. The mitoATP ratio was calculated as (ATP production from mitochondria) / (total cellular ATP production), and the glycolytic ATP ratio was calculated as (ATP production from glycolysis) / (total cellular ATP production).

### MeRIP sequencing

All procedures were performed as we previously described. Total RNA was extracted using Trizol reagent (Invitrogen, CA, USA). The total RNA quality and quantity were analysis of Bioanalyzer 2100 (Agilent, CA, USA) with RIN number >7.0. Approximately more than 25 ug of total RNA representing a specific adipose type was used to deplete ribosomal RNA according to the manuscript of the Epicentre Ribo-Zero Gold Kit (Illumina, San Diego, USA). Following purification, the ribosomal-depleted RNA is fragmented into ∼100-nt-long oligonucleotides using divalent cations under elevated temperature. Then the cleaved RNA fragments were subjected to incubated for 2h at 4°C with m6A-specific antibody (No. 202003, Synaptic Systems, Germany) in IP buffer. The mixture was then incubated with protein-A beads and eluted with elution buffer. Eluted m6A-containing fragments (IP) and untreated input control fragments are converted to final cDNA library in accordance with a strand-specific library preparation by dUTP method. The average insert size for the paired-end libraries was ∼100±50 bp. And then we performed the paired-end 2×150bp sequencing on an Illumina Novaseq™ 6000 platform at the LC-BIO Bio-tech ltd (Hangzhou, China) following the vendor’s recommended protocol.

### Constructs, protein expression and purification

Plasmid encoding YTHDF3 was PCR amplified from the human cDNA. Restriction endonuclease BamHI and XhoI linearize YTHDF3 plasmid, and then ligated into a pET-28a plasmid carrying the Ulp1 cleavage site. Recombinant plasmids were transformed into E. coli BL21 (DE3) to produce target proteins with N-terminal hexahistidine-sumo fusions. Then, E. coli cells were cultured in LB medium until the OD600 reached 0.6–0.8, then 0.2 mM isopropyl-β-D-thiogalactoside (IPTG) was cocultured at 18 °C for 16 hours. Cell extracts were centrifuged for 1 hour at 4 °C and purified with Ni-NTA. Then, the eluted protein was obtained in the buffer containing 10 mM Tris-HCl pH 8.0, 100 mM NaCl. The concentration was determined by A280 and was concentrated to 10 mg/ml.

### Fluorescence recovery after photobleaching (FRAP) experiments in vivo

FRAP was performed on an inverted laser scanning confocal microscope (Zeiss, LSM 800 with airyscan) equipped with an incubation chamber. 488 nm laser line was used for detection of EGFP fluorescence. The point region of YTHDF3-EGFP in cells was chosen and bleached with 5 iterations at ∼60% of maximum laser power at 488 nm. The recovery was recorded at a rate of 30 seconds/interval, 120 cycles in total. To account for photo-bleaching effects during acquisition, we used the mean intensity values from the bleach region, and three independent FRAP experiments were performed in each sample.

### In vitro phase separation assays

For droplet formation, temperature dependent droplet assembly was performed in the following buffer as previously: 20 mM HEPES pH 7.4, 300 mM KCl, 6 mM MgCl2, 0.02% NP-40. For non-fluorescent YTHDF3 (75 µM), droplet-containing buffer was placed on a coverslip and visualized by a phase-contrast using an Olympus IX71 inverted microscope. Temperature-dependent phase separation experiments were performed by incubating YTHDF3 at 37°C after removal from ice.

RNA-dependent droplet-formation experiments were performed in the following buffer: 20 mM HEPES pH 7.4, 300 mM KCl, 6 mM MgCl2, 0.02% NP-40. Non-fluorescent YTHDF3 (25 µM) diluted in buffer was placed on a coverslip and addition of 2μM m6A-modified PPARα (2 μM) was added. The solution was incubated at 37 °C for 10 min and droplets were visualized with phase-contrast microscopy.

### Ultra-high-resolution microscope and HIS-SIM imaging

Super-resolution imaging of Subcellular structures was performed using commercialized HIS-SIM, termed HIS-SIM (High Intelligent and Sensitive SIM) provided by Guangzhou CSR Biotech Co. Ltd. Images were acquired using a 100×/1.5 NA oil immersion objective (Olympus). Cells were seeded in 8-well chambered coverglass and maintained at 37°C and 5% CO2 in a humidified chamber for live SIM imaging. SIM images were collected and analyzed as described previously. Sparse deconvolution was carried out to further improve the image quality.

### Statistical analysis

All statistical analyses in this study were carried out using SPSS 19.0 software. The significance of mean values between two groups was analyzed by Student’s t test (*p < 0.05, **p < 0.01, ***p < 0.001). Pearson correlation analysis was performed to determine the correlation among the indicated protein expression. Pearson’s chi-square test was used to analyze the clinical variables. Kaplan-Meier survival analysis was utilized to compare CRC patient survival based on YTHDF3 expression by log-rank test. p value < 0.05 was considered a significant difference.

## Supplementary Material

pwaf092_Supplementary_Data

## Data Availability

The transcript sequencing data of CRC tissues from clinical patients and the MeRIP sequencing data discussed in this paper have been deposited in NCBI’s Gene Expression Omnibus and are accessible through GEO Series accession numbers GSE129716 and GSE221608. The data will become public when this article is published online. Other data in this study are available from the corresponding author on reasonable request.

## Abbreviations

AOM: azoxymethane; BHB, β-hydroxybutyrate; CHX, cycloheximide; COAD, colon adenocarcinoma; CPT1A, carnitine palmitoyltransferase 1A; CRC, colorectal cancer; CRLM, colorectal cancer liver metastasis; DSS, dextran sulfate sodium; FAO, fatty acid oxidation; FRAP, fluorescence recovery after photobleaching; GEO, Gene Expression Omnibus; GTEx, genotype-tissue expression; HFD, high-fat diet; IHC, immunohistochemistry; Kbhb, lysine β-hydroxybutyrylation; LDs, lipid droplets; LLPS, liquid-liquid phase separation; MeRIP, methylated RNA immunoprecipitation; m6A, N6-methyladenosine; NAFLD, nonalcoholic fatty liver disease; OA, oleic acid; OPLS-DA, orthogonal partial least-squares discriminant analysis; PA, palmitic acid; PDOs, patient-derived organoids; PPARα, peroxisome proliferator-activated receptor alpha; SC, standard chow; STAT3, signal transducer and activator of transcription 3; TCGA, The Cancer Genome Atlas; TRF, time-restricted feeding; YAP, yes-associated protein; YTHDF3, YTH domain-containing family protein 3
