# Supplementary material for: Lipid deposition promotes YTHDF3-mediated m6A modification of PPARα to facilitate liver metastasis of colorectal cancer
Source: Protein Cell. 2025 Nov 5;17(3):190–211. doi: 10.1093/procel/pwaf092 (PMC12987569; doi:10.1093/procel/pwaf092)
Supplement: pwaf092_Supplementary_Data [file pwaf092_supplementary_data.zip › PAC-25420-LJM-SUPPORTING INFORMATION.pdf]

METHODS

Data availability statement

The transcript sequencing data of CRC tissues from clinical patients and the MeRIP sequencing data discussed in this paper have been deposited in NCBI's Gene Expression Omnibus and accessible through GEO Series accession number GSE129716 and GSE221608. The data will become public when this article is published online. Other data in this study are available from the corresponding author on reasonable request.

Tissue samples and immunohistochemistry (IHC) staining

All matched tissue samples were collected simultaneously from the same patient during a single surgical operation. Specifically, normal intestinal mucosa (Nor) was obtained from histologically confirmed tumor-free margins (>2 cm from the primary tumor lesion). Primary colorectal cancer (CRC) tissue (Ca) was derived from the primary tumor site, while liver metastasis (LM) samples were obtained from synchronous metastatic lesions in the liver. None of the patients received neoadjuvant chemotherapy or radiotherapy prior to surgery. Detailed patient information, including surgical site and pathological staging, is summarized in Table S5. The median time interval between the resection of the primary tumor and the corresponding liver metastasis was less than two hours. Formalin-fixed paraffin-embedded (FFPE) CRC tissues and adjacent normal tissues were collected from the Department of Pathology at Sun Yat-sen Memorial Hospital, Sun Yat-sen University (Guangzhou, China). The study was approved by the Ethics Committees of Sun Yat-sen University with Approval No. SYSKY-2023-012-01. IHC was performed as we previously described. And IHC staining scores were evaluated in a blinded fashion. The scoring system from 0 to 12 combined the intensity and percentage (signal: "0," no staining; "1," weak staining; "2," intermediate staining; and "3," strong staining; percentage: "0," 0%; "1," 1%–25%; "2," 26%–50%; "3," 51%–75%; "4," >75%), which were used as we described previously. The median value of total staining scores was identified as the optimal cut-off value.

Tumor dataset acquisition and process

The datasets and corresponding clinical data presented in our study were downloaded from the Cancer Genome Atlas (TCGA) and Genotype-Tissue Expression (GTEx) data portal. Tumor RNA-seq data can be downloaded from the Genomic Data Commons (GDC) data portal website. We used R software to calculate the difference expression between normal and tumor samples in each tumor. Univariate cox regression analysis and forest plots through the "forestplot" R package was used to display the P value, HR and 95% CI of each variable. R software (version 4.0.3) was used for statistical analysis. If not otherwise stated, unpaired Wilcoxon rank sum and signed rank tests were used for significance analysis, the asterisk represents the degree of significance (*p), and P value <0.05 is considered statistically significant.

Animal models

The animal studies were conducted in accordance with the guidelines approved by the Institutional Animal Care and Use Committee of Sun Yat-sen University with Approval No.SYSU-IACUC-2022-B0790. C57BL/6 mice were purchased from Cyagen

Biosciences Inc., and Ythdf3 knockout (Ythdf3−/−) mice, in which exon 3 of Ythdf3 gene was deleted by a CRISPR/ Cas9 System, were established in our lab. All mice were maintained under SPF conditions of Sun Yat-sen University Animal Center. The azoxymethane (AOM) and dextran sulfate sodium (DSS) model of colon carcinoma has been described previously. 6–8-week-old mice were injected with 10mg/kg body weight AOM once a week for three times. And mice were typically subjected to three cycles of weekly 1% DSS exposures, each followed by a 2-week rest period. The animals were sacrificed 20 weeks to measure the number and incidence of tumors. For xenografts tumor model, 2 X 10^6 cells were subcutaneously injected into nude mice (BALB/c, SPF grade, 6–8 weeks old, n = 6 per group). And tumor sizes were monitored every 3 days for 3 weeks. For in vivo pulmonary metastasis, 1 x $10^{\wedge 6}$ CRC cells were injected via the tail vein, leading to lung metastasis within 4–6 weeks (with a success rate of ≥ 99%). The intrasplenic injection liver metastasis model involves a caudal spleen injection followed by a hemi-splenectomy, resulting in liver metastasis within 4–6 weeks (with a success rate of ≥ 99%). These models are characterized by a high success rate (with over 99% of mice developing metastases), a short experimental timeline (4–6 weeks), and reproducibility and standardization, making them ideal for studying metastatic colonization and therapeutic interventions. For constructing a time-restricted feeding (TRF) model in mice, we choose a duration of 8 hours (from 7 PM to 3 AM) as feeding window to mimic the benefits of intermittent fasting. Then animals were sacrificed 45 days and the ratio of tumor metastasis loci was calculated.

Construction of patient-derived organoids (PDOs) models

The study received approval from the Human Research Ethics Committees of Sun Yat-sen University and adheres to all relevant ethical regulations for human research participants. Informed consent was obtained from all subjects. To construct PDOs, we collected tumor specimens from patients with colorectal cancer with separate liver metastases and cultured tumor tissues in a specialized medium that promotes the growth of organoid structures (Precedo, China, Cat.PRS-ICM-3D). The sample should be collected in a sterile container and transported to the laboratory as soon as possible. The tumor tissue is dissociated into single cells, which are embedded in a matrix and plated in a 3D culture system (Precedo, China, Cat. PRS-LM5). The cells are cultured in a humidified incubator at 37°C with 5% CO_2. Over time, the cells self-organize and form three-dimensional structures that resemble the original tissue or organ. The organoids can be passaged and expanded for further experiments. HE and IHC staining of PDOs were used to identify of CRC tissues. The data will become public when this article is published online. The study received approval from the Human Research Ethics Committees of Sun Yat-sen University and adheres to all relevant ethical regulations for human research participants. Informed consent was obtained from all subjects.

Widely-targeted lipidomic analysis and ELISA assay

For Widely-targeted lipidomic analysis, sample was thawed on ice and taken 20 mg of one sample and homogenized it with 1mL mixture (include methanol, MTBE and internal standard mixture) and steel ball. Take out the steel ball and whirl the mixture for 15min. Add 200 uL of water and whirl the mixture for 1 min, and then centrifuge it

with 12,000 rpm at 4 °C for 10 min. Extract 300 uL supernatant and concentrate it. Dissolve powder with 200 uL mobile phase B, then stored in -80 °C. Finally take the dissolving solution into the sample bottle for LC-MS/MS analysis. Significantly regulated metabolites between groups were determined by VIP≥ 1 and absolute Log2FC (fold change) ≥1. VIP values were extracted from OPLS-DA result, which also contain score plots and permutation plots, was generated using R package MetaboAnalystR. The data was log transform (log2) and mean centering before OPLS-DA. In order to avoid overfitting, a permutation test (200 permutations) was performed.

For ELISA assay, after cutting specimens, weigh 1 g of tissue and homogenize the specimens adequately by homogenizer. Centrifuge for about 20 minutes (2000 rpm) and carefully collect the supernatant. Pack one part to be tested and the rest to be frozen for reserve. Then add standard sample diluent and incubate for 30minutes at 37° C. Add HRP-Conjugate reagent and incubate for 30 minutes at 37° C. Then add chromogen solution A and B and incubate for 10 minutes at 37° C. Finally, add stop solution and read absorbance at 450nm within 15minutes.

Fatty acid oxidation (FAO) and Seahorse Real-Time ATP Rate assay

Fatty acid oxidation (FAO) activity was measured using the FAO Assay Kit (Assay Genie, Dublin, Ireland; Cat# BR00001) following the manufacturer's instructions. Briefly, cells were lysed on ice for 5 minutes, then centrifuged at 14,000 rpm for 5 minutes, and the supernatant was collected. Protein concentration was determined using the BCA assay. Equal amounts of protein (typically 10–20 μg) were added to a 96-well plate in duplicate. Control solution (without substrate) and reaction solution (containing 20× FAO substrate, octanoyl-CoA) were added to respective wells. After incubation at 37°C for 30–120 minutes, the reaction was stopped by adding 3% acetic acid. The absorbance was measured at 492 nm using a microplate reader. FAO activity was calculated by subtracting the control well absorbance from the reaction well absorbance. This assay detects NADH generation coupled with the reduction of INT (a tetrazolium salt), forming a red formazan product proportional to FAO activity. Octanoyl-CoA is used as a medium-chain fatty acid substrate to ensure solubility and mitochondrial enzyme accessibility.

The Seahorse Real-Time ATP Rate Assay is performed according with the manufacturer’s instructions (Agilent, California, USA). Firstly, prepare assay media and Seahorse XF cell culture microplate for assay. Then, metabolic modulators (oligomycin and a mix of rotenone and antimycin A) were serially injected, allow the calculation of the mitochondrial and glycolytic ATP production rates. After running assay, using the Agilent Seahorse XF Real-Time ATP Rate Assay Report Generator to calculates the XF Real-Time ATP Rate Assay Parameters (mitoATP Production Rate, glycoATP Production Rate, total ATP Production Rate, XF ATP Rate Index, % glycolysis, and % OXPHOS). The quantification of the "glycolytic ATP ratio" and "mitoATP ratio" was conducted using the Agilent Seahorse XF Real-Time ATP Rate Assay Kit (Cat# 103592-100), following the manufacturer's protocol. Baseline

measurements of oxygen consumption rate (OCR) and extracellular acidification rate (ECAR) were taken before the injection of oligomycin (1 μM) to inhibit mitochondrial ATP production. Subsequently, rotenone and antimycin A (0.5 μM each) were administered to halt mitochondrial respiration. The values were automatically computed using Agilent Seahorse Wave Desktop v2.6 software and presented as the mean ± standard deviation (SD) from four separate experiments, with five wells per group. The mitoATP ratio was calculated as (ATP production from mitochondria) / (total cellular ATP production), and the glycolytic ATP ratio was calculated as (ATP production from glycolysis) / (total cellular ATP production).

MeRIP sequencing

All procedures were performed as we previously described. Total RNA was extracted using Trizol reagent (Invitrogen, CA, USA). The total RNA quality and quantity were analysis of Bioanalyzer 2100 (Agilent, CA, USA) with RIN number >7.0. Approximately more than 25 ug of total RNA representing a specific adipose type was used to deplete ribosomal RNA according to the manuscript of the Epicentre Ribo-Zero Gold Kit (Illumina, San Diego, USA). Following purification, the ribosomal-depleted RNA is fragmented into ~100-nt-long oligonucleotides using divalent cations under elevated temperature. Then the cleaved RNA fragments were subjected to incubated for 2h at 4°C with m6A-specific antibody (No. 202003, Synaptic Systems, Germany) in IP buffer. The mixture was then incubated with protein-A beads and eluted with elution buffer. Eluted m6A-containing fragments (IP) and untreated input control fragments are converted to final cDNA library in accordance with a strand-specific library preparation by dUTP method. The average insert size for the paired-end libraries was ~100±50 bp. And then we performed the paired-end 2×150bp sequencing on an Illumina Novaseq™ 6000 platform at the LC-BIO Bio-tech ltd (Hangzhou, China) following the vendor's recommended protocol.

Constructs, protein expression and purification

Plasmid encoding YTHDF3 was PCR amplified from the human cDNA. Restriction endonuclease BamHI and XhoI linearize YTHDF3 plasmid, and then ligated into a pET-28a plasmid carrying the Ulp1 cleavage site. Recombinant plasmids were transformed into E. coli BL21 (DE3) to produce target proteins with N-terminal hexahistidine-sumo fusions. Then, E. coli cells were cultured in LB medium until the OD_{600} reached 0.6–0.8, then 0.2 mM isopropyl-β-D-thiogalactoside (IPTG) was cocultured at 18 °C for 16 hours. Cell extracts were centrifuged for 1 hour at 4 °C and purified with Ni-NTA. Then, the eluted protein was obtained in the buffer containing 10 mM Tris-HCl pH 8.0, 100 mM NaCl. The concentration was determined by A280 and was concentrated to 10 mg/ml.

Fluorescence recovery after photobleaching (FRAP) experiments in vivo

FRAP was performed on an inverted laser scanning confocal microscope (Zeiss, LSM 800 with airyscan) equipped with an incubation chamber. 488 nm laser line was used for detection of EGFP fluorescence. The point region of YTHDF3-EGFP in cells was chosen and bleached with 5 iterations at ~60% of maximum laser power at 488 nm. The recovery was recorded at a rate of 30 seconds/interval, 120 cycles in total. To account for photo-bleaching effects during acquisition, we used the mean intensity values from

the bleach region, and three independent FRAP experiments were performed in each sample.

In vitro phase separation assays

For droplet formation, temperature dependent droplet assembly was performed in the following buffer as previously: 20 mM HEPES pH 7.4, 300 mM KCl, 6 mM MgCl2, 0.02% NP-40. For non-fluorescent YTHDF3 (75 µM), droplet-containing buffer was placed on a coverslip and visualized by a phase-contrast using an Olympus IX71 inverted microscope. Temperature-dependent phase separation experiments were performed by incubating YTHDF3 at 37 °C after removal from ice.

RNA-dependent droplet-formation experiments were performed in the following buffer: 20 mM HEPES pH 7.4, 300 mM KCl, 6 mM MgCl2, 0.02% NP-40. Non-fluorescent YTHDF3 (25 µM) diluted in buffer was placed on a coverslip and addition of 2µM m^6A-modified PPARα (2 µM) was added. The solution was incubated at 37 °C for 10 min and droplets were visualized with phase-contrast microscopy.

Ultra-high-resolution microscope and HIS-SIM imaging

Super-resolution imaging of Subcellular structures was performed using commercialized HIS-SIM, termed HIS-SIM (High Intelligent and Sensitive SIM) provided by Guangzhou CSR Biotech Co. Ltd. Images were acquired using a 100×/1.5 NA oil immersion objective (Olympus). Cells were seeded in 8-well chambered coverglass and maintained at 37℃ and 5% CO2 in a humidified chamber for live SIM imaging. SIM images were collected and analyzed as described previously. Sparse deconvolution was carried out to further improve the image quality.

Statistical analysis

All statistical analyses in this study were carried out using SPSS 19.0 software. The significance of mean values between two groups was analyzed by Student's t test (*$p < 0.05$, **$p < 0.01$, ***$p < 0.001$). Pearson correlation analysis was performed to determine the correlation among the indicated protein expression. Pearson's chi-square test was used to analyze the clinical variables. Kaplan-Meier survival analysis was utilized to compare CRC patient survival based on YTHDF3 expression by log-rank test. p value < 0.05 was considered a significant difference.

SUPPORTING INFORMATION

Supporting Information is available Online, including:

- Fig.s S1-S16.
- Key resources table
- Table S1-S6. Excel file containing additional data too large to fit in PDF.
- Video S1. Interaction of YTHDF3 and lipid droplets in CRC cells treated with OA.
- Key resources table

SUPPORTING INFORMATION

Supporting Information is available Online, including:

- Figures S1-S16.
- Key resources table
- Table S1. Differential lipid metabolite profiles in normal intestinal mucosa, primary colorectal cancer tissues, and liver metastases from CRC patients (n=24)
- Table S2. The spatial distribution of differentially lipid metabolites profiles of tumors in high fat diet versus standard chow groups (n=4)
- Table S3. The spatial distribution of differentially lipid metabolites showed that Ythdf3 knockout abrogated the HFD-mediated lipids deposition in tumors (n=4)
- Table S4. Differentially expressed genes both in transcriptomic and MeRIP sequencing in HCT116 cells transfected with YTHDF3 siRNA
- Table S5. Information of CRC patients by widely-targeted lipidomics analysis (n=24)
- Table S6. LC-MS analysis of Kbhb modificaiton proteins in CRC cells treated with BHB.
- Video S1. Interaction of YTHDF3 and lipid droplets in CRC cells treated with OA.

Lipid deposition promotes YTHDF3-mediated m^6A modification of PPARα to facilitate liver metastasis of colorectal cancer

Wen Ni[1,2, #], Yuanyuan Xu[1,2, #], Mengrou Zhang[1,2, #], Yuqing Li[1,2, #], Piao Huang[1,2 #], Zhun Li [1,2], Qi Wu [1,2], Hui Mo[1,2], Yibiao Ye[3], Yuhui Li[1,2], Aijun Zhou[1], Su Yao[6], Shilin Zhi [1,2], Jiali Qi [1,2], Shuhui Yu[1,2], Saiqi He[1,2], Jianming Li[1,2,4, 5*]

[1] Department of Pathology, Sun Yat-sen Memorial Hospital, Sun Yat-sen University, Guangzhou 510120, China;

[2] Guangdong Provincial Key Laboratory of Malignant Tumor Epigenetics and Gene Regulation, Sun Yat-sen Memorial Hospital, Sun Yat-sen University, Guangzhou 510120, China;

[3] Department of Hepato-Billiary Surgery, Sun Yat-sen Memorial Hospital, Sun Yat-sen University, Guangzhou 510120, China;

[4] The MOE Basic Research and Innovation Center for the Targeted Therapeutics of Solid Tumors, The First Affiliated Hospital, Jiangxi Medical College, Nanchang University, Nanchang 330006, China

[5] Department of Pathology and Institute of Molecular Pathology, The First Affiliated Hospital, Jiangxi Medical College, Nanchang University, Nanchang 330006, China

[6]Department of Pathology, Guangdong Provincial People's Hospital, Guangdong

Academy of Medical Sciences, Southern Medical University, Guangzhou 510080, China

#These authors contributed equally

* **Correspondence to:**

Jian-Ming Li, Department of Pathology, Sun Yat-sen Memorial Hospital, Sun Yat-sen University, Guangzhou, China, Phone: +86 20 81332590; Fax: +86 20 81332590; E-mail: lijming3@mail.sysu.edu.cn

ORCID: 0000-0002-2862-9922

Keywords: Colorectal Cancer; Liver Metastasis; Lipid Metabolism Reprograming; m^6A Modification; β-hydroxybutyrylation; Metabolites

SUPPORTING FIGURES AND FIGURE LEGENDS

Fig. S1

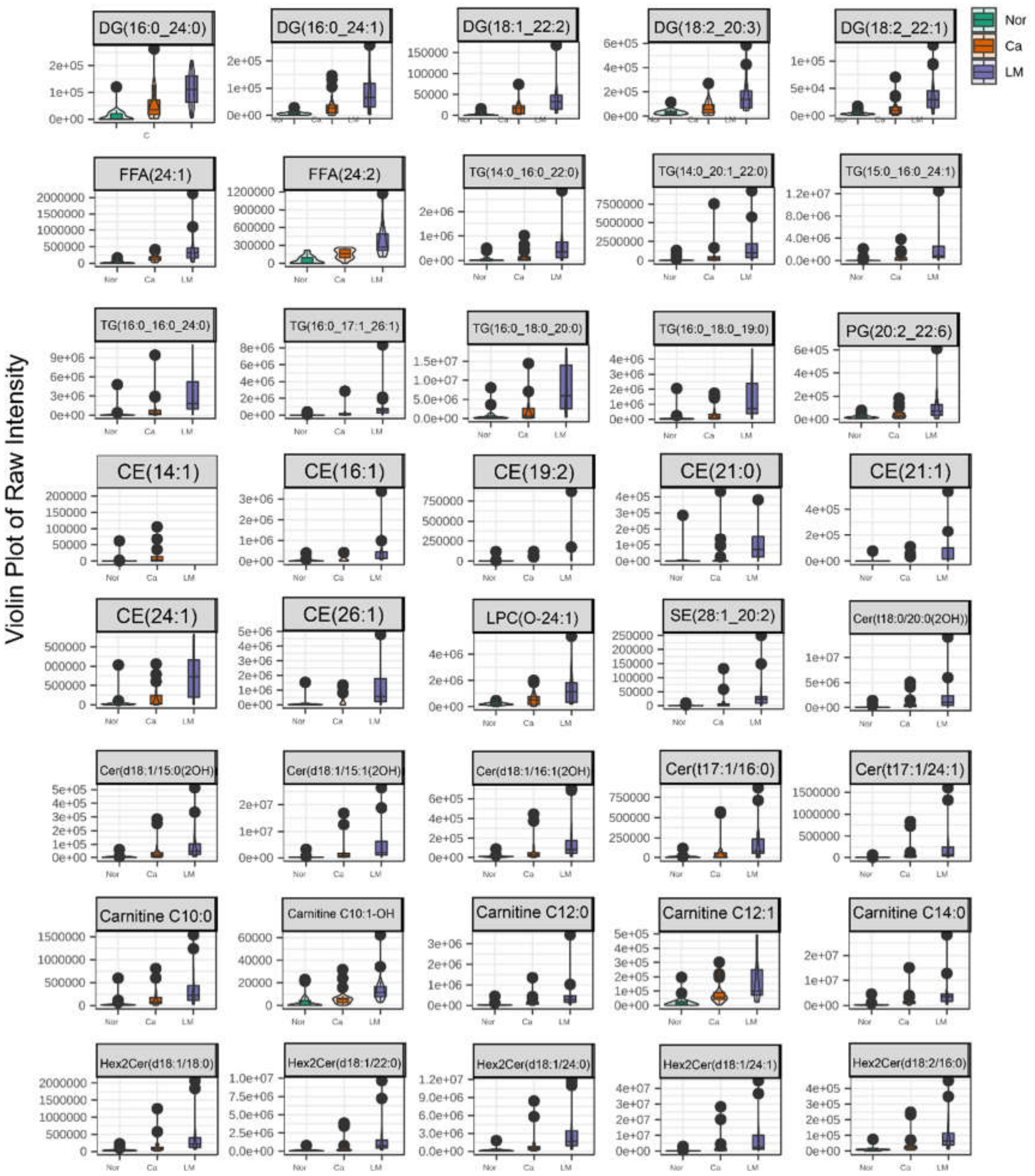

Figure S1. Violin plot of differential lipids metabolites in CRCLM tissues. The abscissa is for grouping, and the ordinate is the relative content of differential lipids (original peak area).

Fig. S2

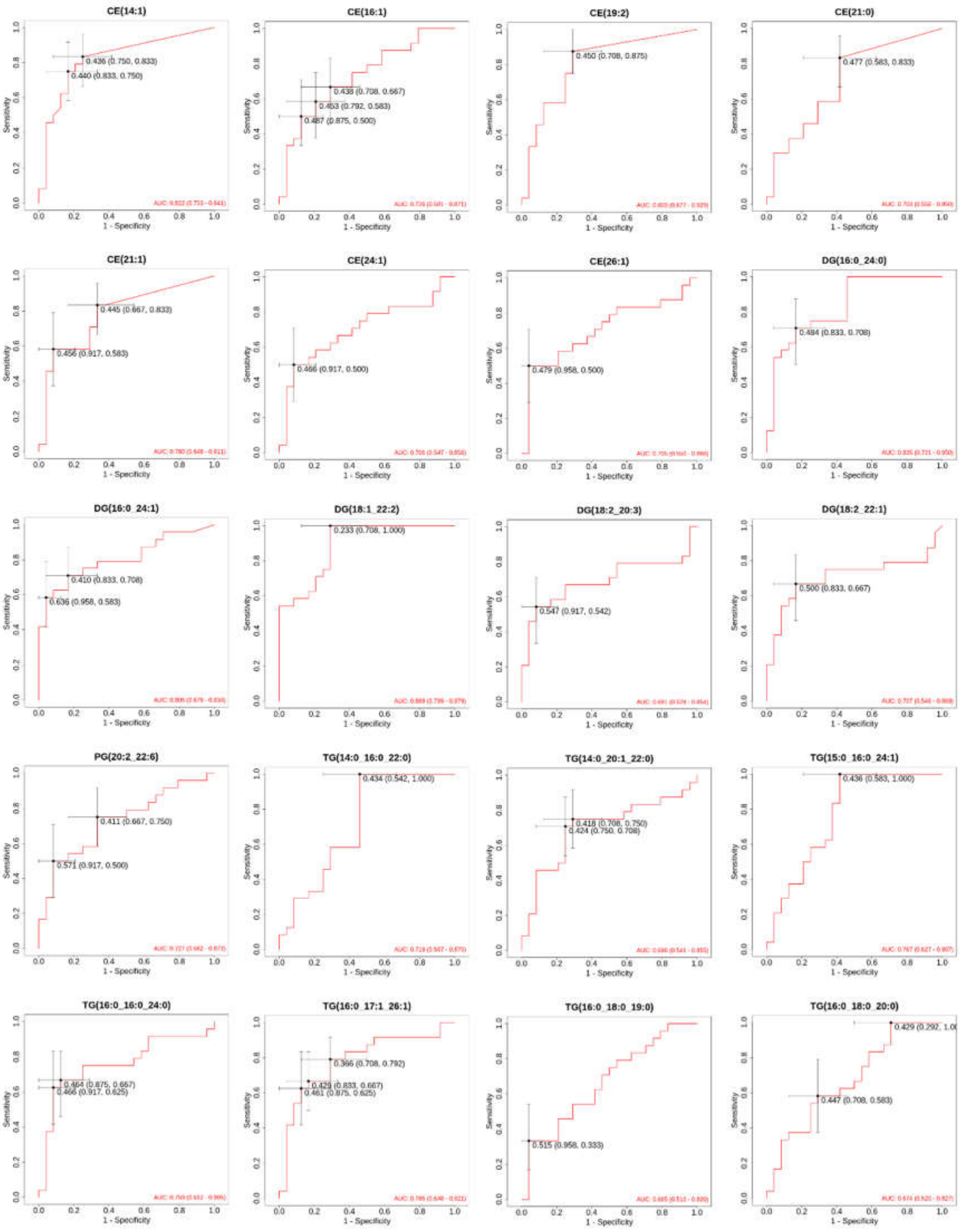

Figure S2. The AUC curve of 20 differential lipid metabolites with an AUC score greater than 0.65 in the primary CRC tumor group (PT) relative to the normal intestinal mucosa group (NC);

Fig. S3

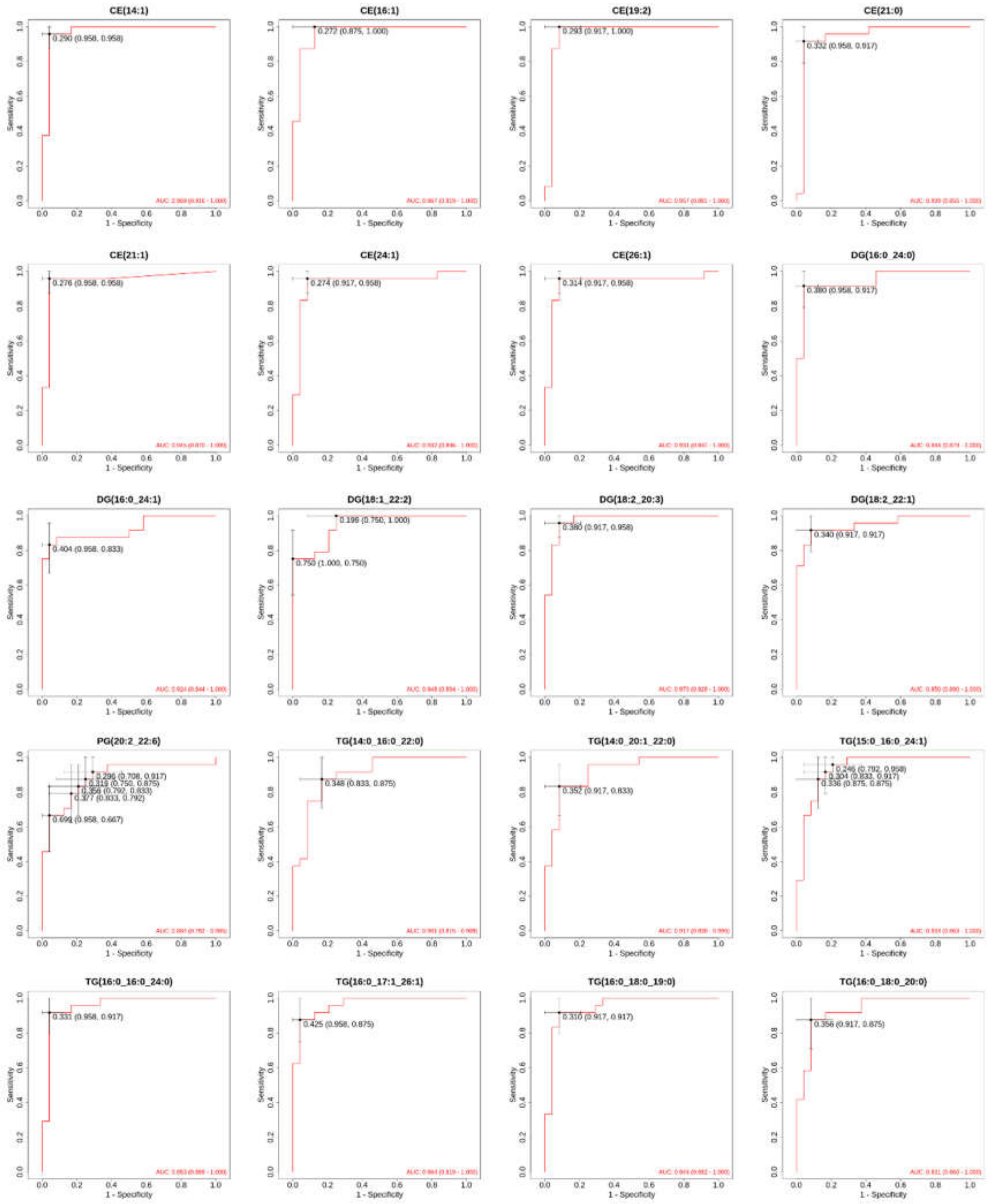

Figure S3. The AUC curve of 20 differential lipid metabolites with an AUC score greater than 0.9 in the colorectal cancer liver metastasis tumor group (TM) relative to the normal intestinal mucosa group (NC).

Fig. S4

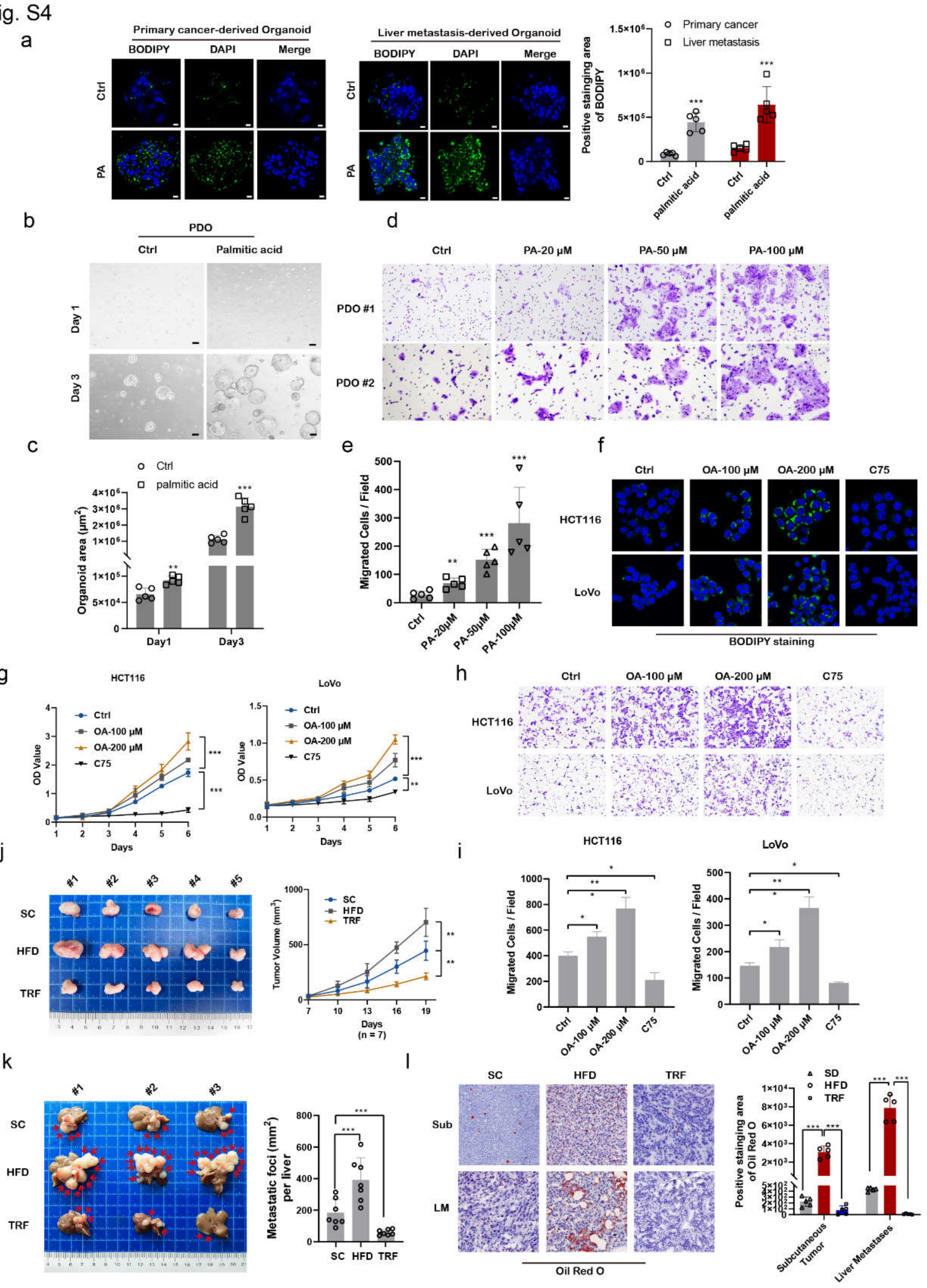

Figure S4. Lipid accumulation enhances CRC cell proliferation and metastasis in *vitro* and in *vivo*

a. Lipids staining by BODIPY 493/503 in the PDOs with PA treated of primary tumor and liver metastases. The ruler represents a scale of 25 μm. ***, $p < 0.001$

b-e. PA enhanced the proliferation (b-c) and invasion (d-e) potential of PDOs in *vitro*. The ruler represents a scale of 25 μm. ***, $p < 0.001$

f. Comparison of lipids deposition in the indicated CRC cells by BODIPY.

g-i. proliferation (g) and invasion (h-i) potential of CRC cells treated with OA or C75. All experiments were performed in triplicate, and results are presented as mean ± SD. ***$P < 0.001$, **$P < 0.01$, *$P < 0.05$.

j. Subcutaneous xenograft model indicated HFD enhances CRC tumor progression. While TRF yields the opposite results.

k. Mice splenic injection of liver metastases model indicated HFD enhances metastasis and promotes expression of YTHDF3 in liver metastases foci. Whereas, TRF yields the opposite results.

l. The Oil Red O staining demonstrates LDs deposition in the indicated groups of mice models.

Fig. S5

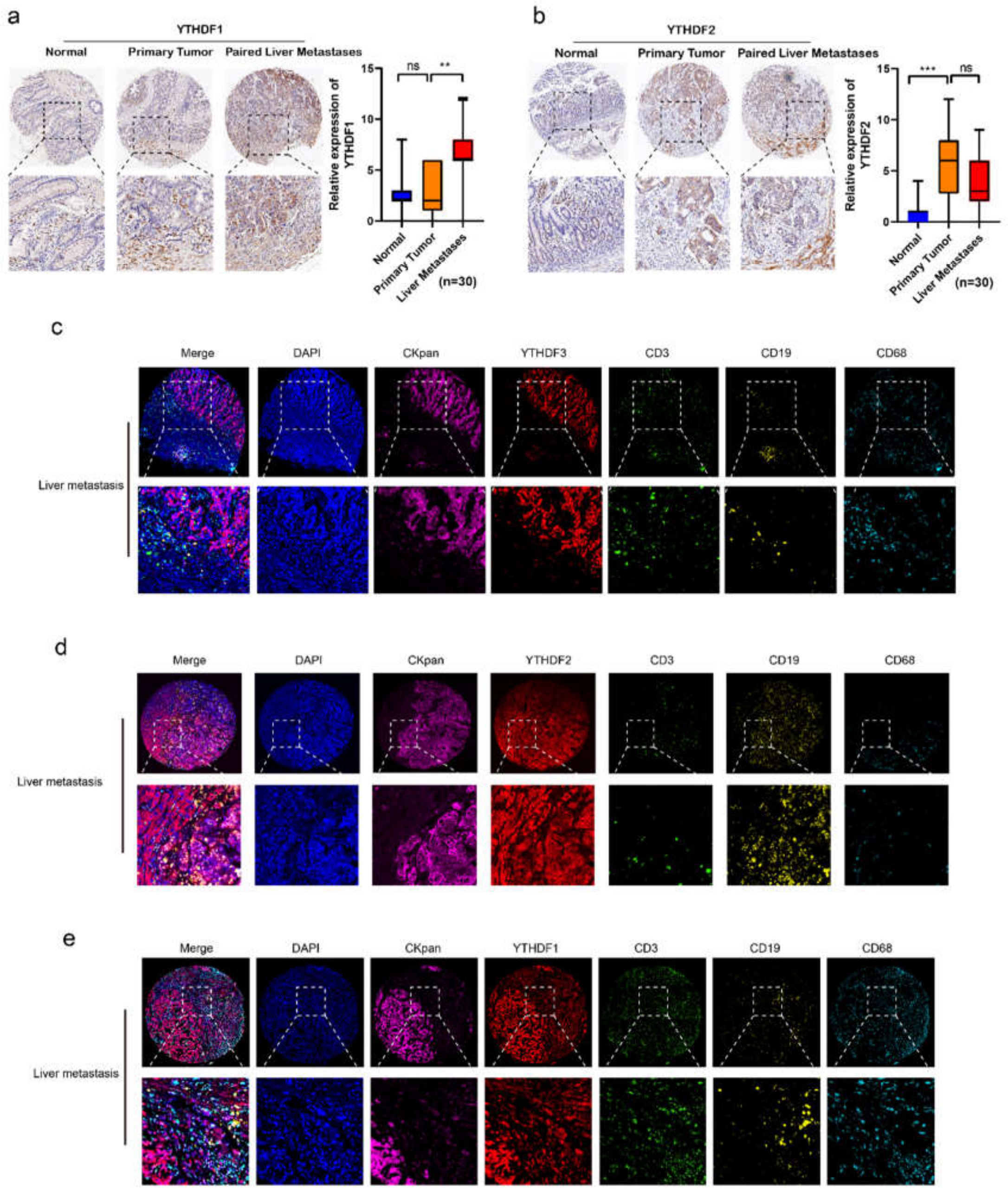

Figure S5. The expression of YTH domain-containing family protein in normal intestinal mucosal tissues, matched primary CRC tissues and liver metastases lesions

a-b. IHC showed the levels of YTHDF1 (a) and YTHDF2 (b) in normal intestinal mucosal tissues, matched primary CRC tissues and liver metastases lesions.

c-e. Multiplexed immunofluorescence staining showed the expression of YTHDF3 (c),

YTHDF1 (d) and YTHDF2 (e) in tumor cells or tumor stromal cells. CKpan labeled epithelial cells, CD3 labeled T cells, CD19 labeled B cells and CD68 labeled macrophages.

Fig. S6

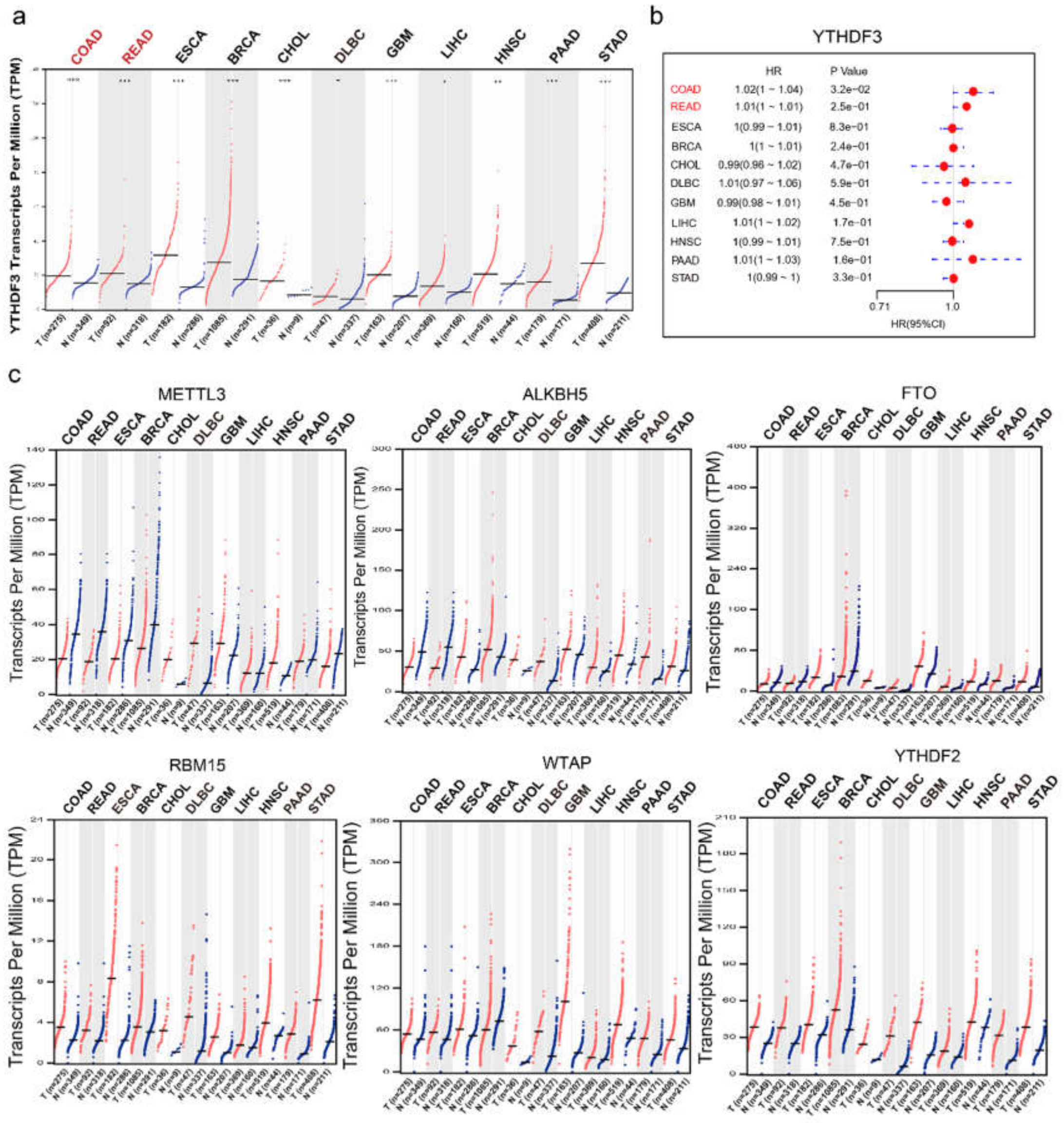

Figure S6. The expression pattern of candidate m^{6}A RNA methylation regulatory factors in TCGA database and the GTEx projects.

a. The expression distribution of YTHDF3 in tumor tissues and normal tissues in TCGA

and GTEx database. The horizontal axis represents different tumor tissues, and the vertical axis represents YTHDF3 expression distribution. (*$p < 0.05$, **$p < 0.01$, ***$p < 0.001$)

b. Forest plots to display the P value, Hazard ratio and 95% CI of YTHDF3 in related cancers.

c. The expression distribution of other m^{6}A RNA methylation regulatory factors in tumor tissues and normal tissues in TCGA and GTEx database.

Fig. S7

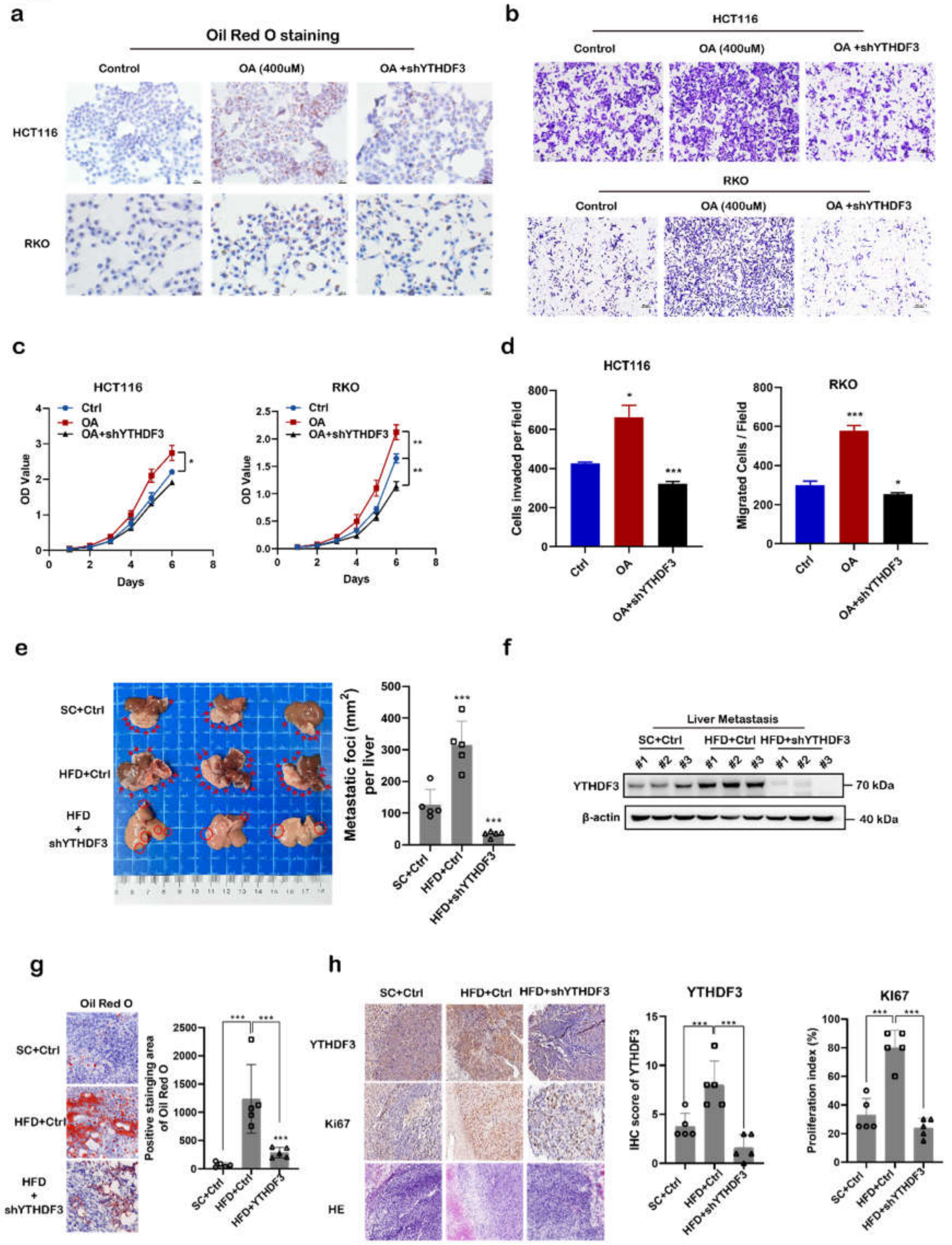

Figure S7. Knockdown of YTHDF3 dampened lipid deposition- induced CRC cells proliferation and metastasis

a. Oil red O staining of indicated CRC cells.

b-d. proliferation (c) and invasion (b,d) potential of CRC cells in the indicated groups.

All experiments were performed in triplicate, and results are presented as mean ± SD. ***P < 0.001, **P < 0.01, *P < 0.05.

e-f. The liver metastasis models of mice achieved the results that knockdown of YTHDF3 declined WD model-induced liver metastasis of tumor.

g. Oil red O staining of indicated groups.

h. HE and IHC staining of indicated proteins.

Fig. S8

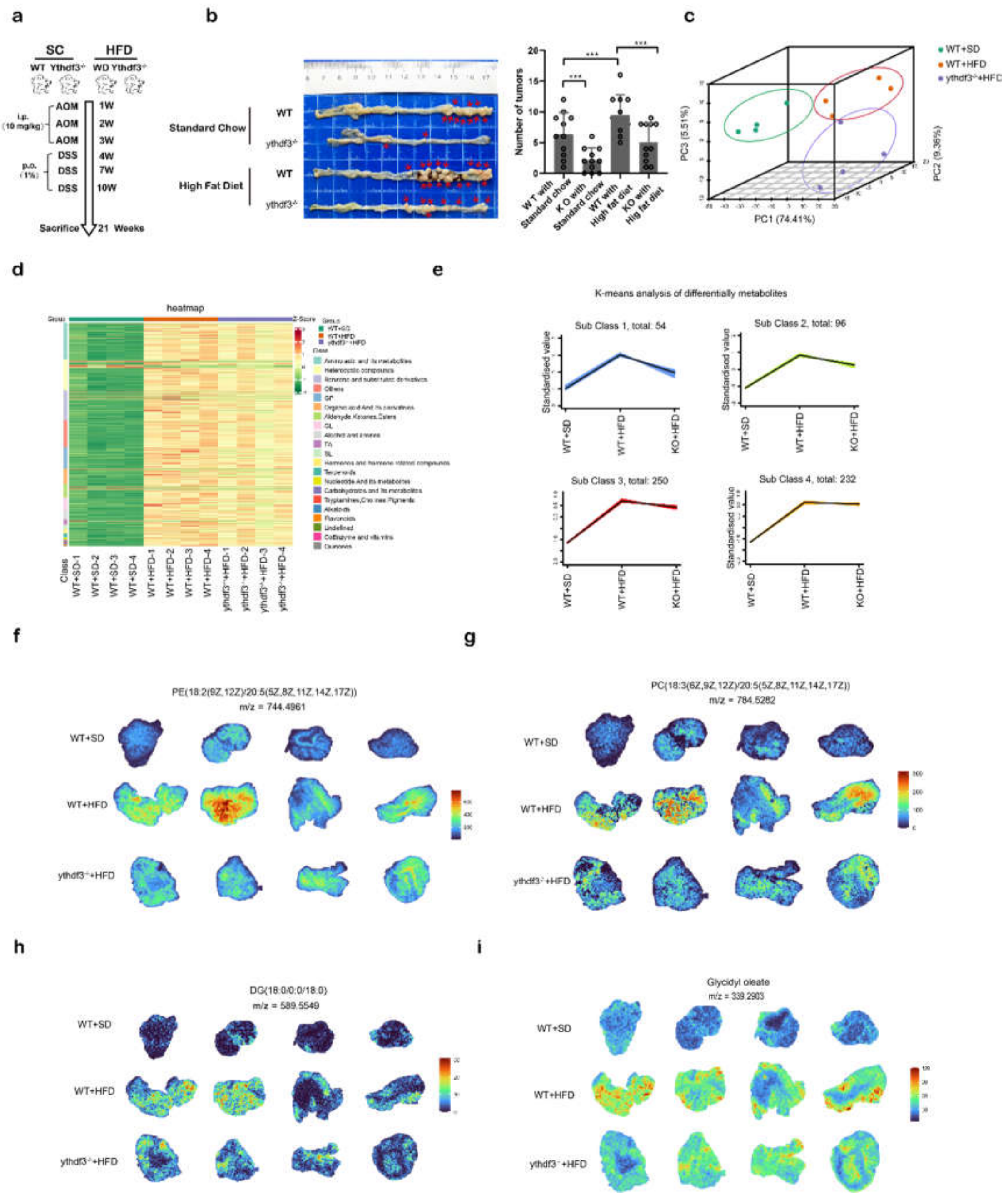

Figure S8. AOM and DSS inducible model of colon carcinoma results showed Ythdf3 knockout attenuates WD induced CRC tumor progression

a. Construction of the Western diet model in the AOM/DSS-induced colorectal cancer model.

b. Ythdf3 knockout attenuates WD induced CRC tumor progression.

c-d. PCA (c) and heatmap plots (d) indicated differentially metabolites profiles of

indicated groups.

e. K-means clustering of differential metabolites of indicated groups.

f-i. The spatial distribution of differentially lipid metabolites in tumor tissues of indicated groups.

Fig. S9

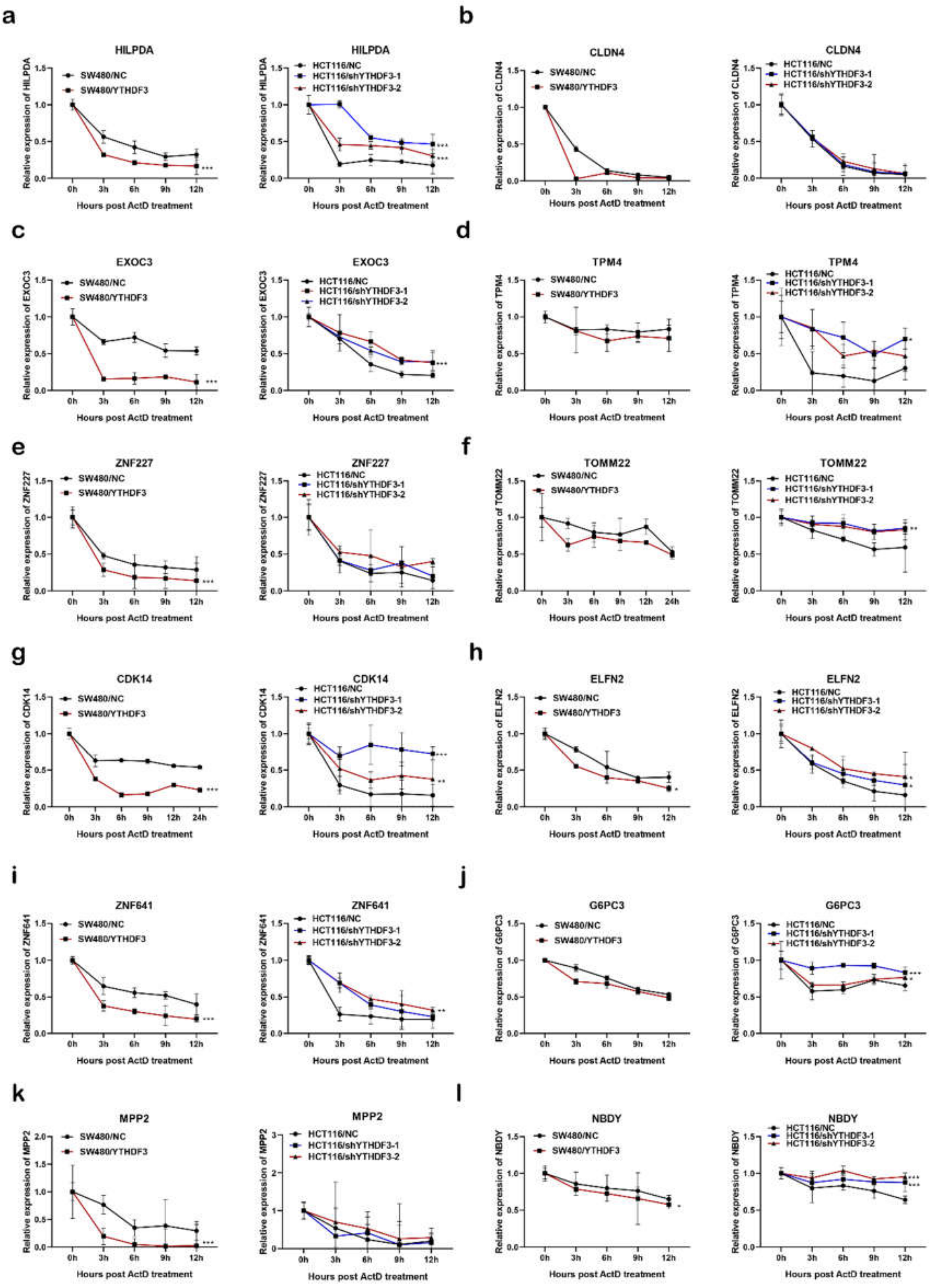

Figure S9. RNA life-time profiling analysis of indicated m^6A-containing mRNAs

Fig. S10

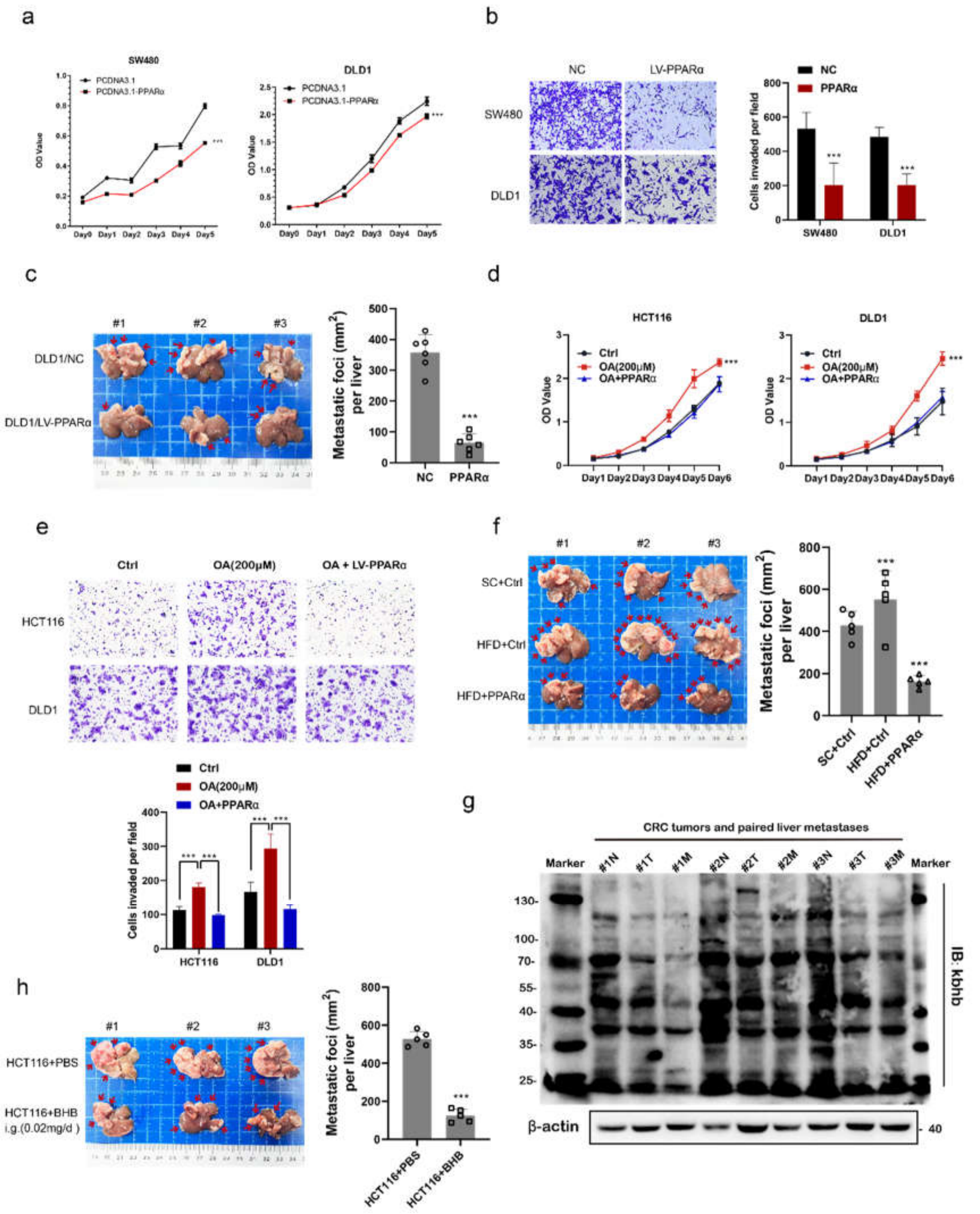

Figure S10. PPARα is a key regulator for CRC liver metastasis

a-b. PPARα overexpression inhibits CRC cell proliferation (a) and invasion (b) in vitro. ***, $p < 0.001$

c. PPARα overexpression inhibits liver metastasis of CRC cells in the mouse splenic injection model. ***, $p < 0.001$

d-f. PPARα overexpression reverses the promotion of CRC cell proliferation and metastasis induced by lipid accumulation in vitro (d-e) and in vivo(f).

g. Kbhb modification was declined in primary CRC tissues and paired liver metastases compared with paired normal tissues.

h. Liver metastasis model of CRC cells in mice identified that BHB intragastric administration declined liver metastases of CRC cells in *vivo.*

Fig. S11

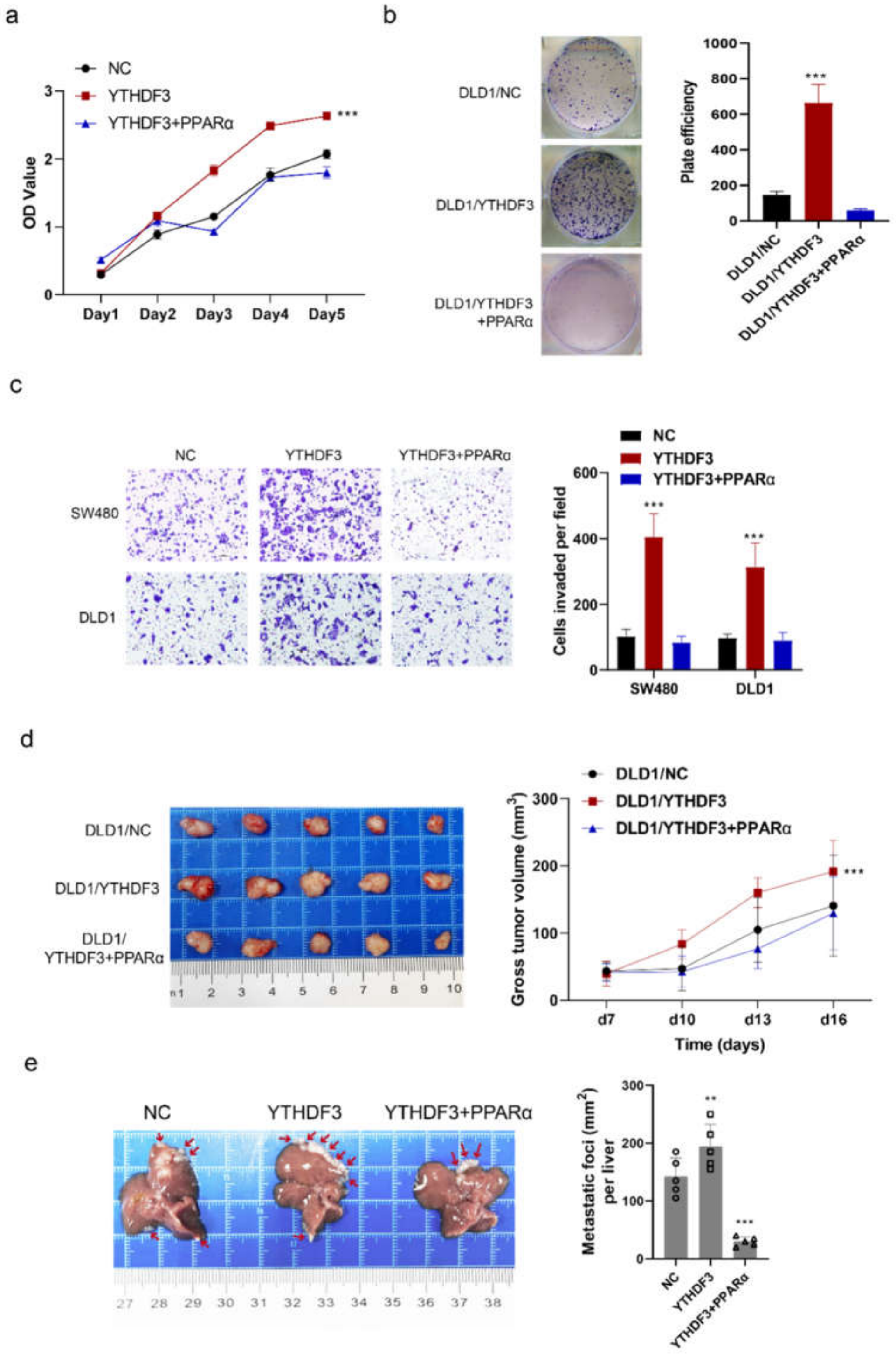

Figure S11. PPARα attenuated YTHDF3-mediated CRC cell proliferation and metastasis in *vitro* and in *vivo*

a-e. PPARα reversed YTHDF3-mediated promotion of CRC cell proliferation and metastasis in vitro (a-c) and in vivo (d-e). ***$P < 0.001$, **$P < 0.01$

Fig. S12

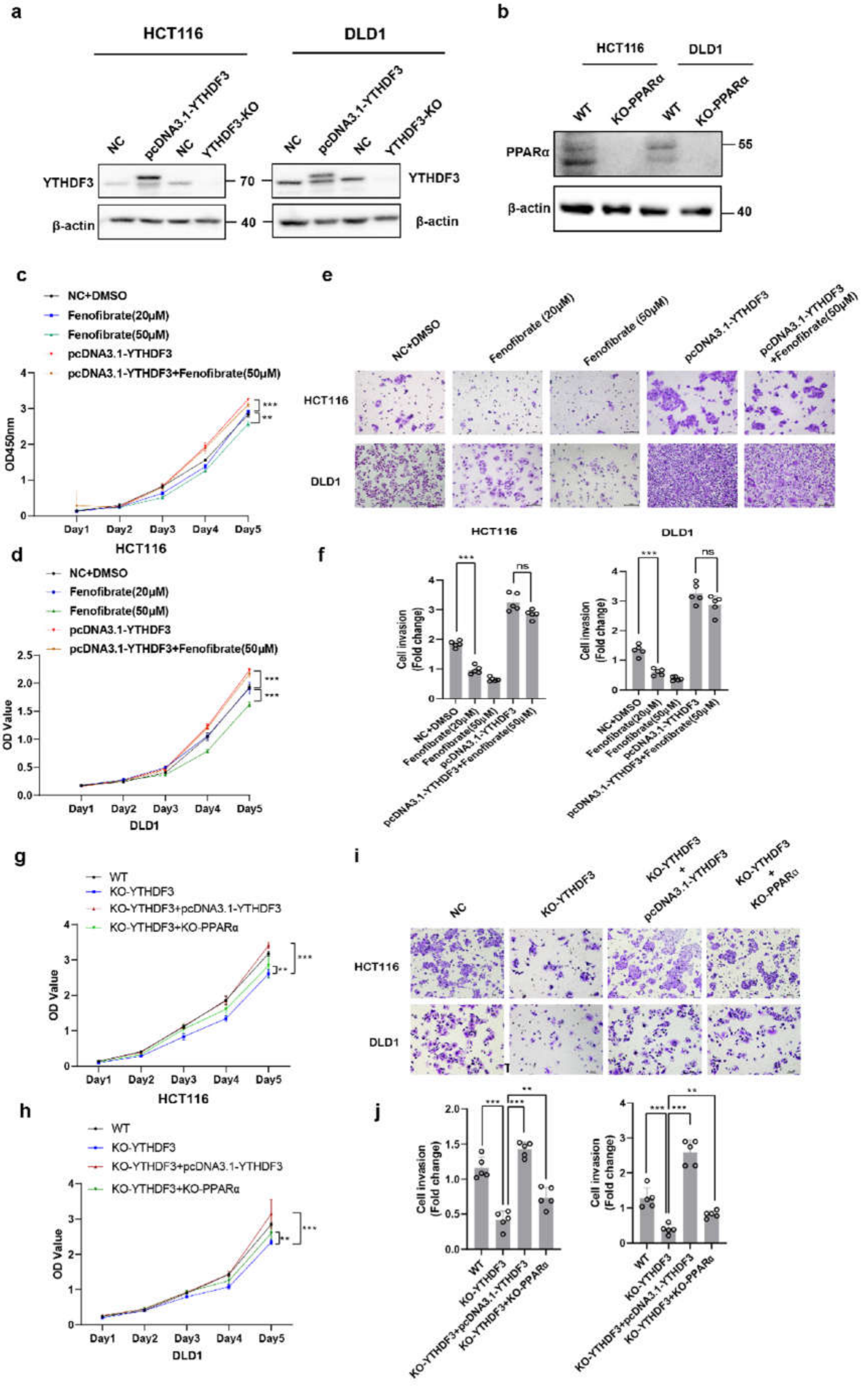

a
HCT116
DLD1
NC
pcDNA3.1-YTHDF3
NC
YTHDF3-KO
YTHDF3
β-actin
70
40
b
HCT116
DLD1
WT
KO-PPARα
PPARα
β-actin
55
40
c
NC+DMSO
Fenofibrate(20μM)
Fenofibrate(50μM)
pcDNA3.1-YTHDF3
pcDNA3.1-YTHDF3+Fenofibrate(50μM)
OD450nm
Day1
Day2
Day3
Day4
Day5
HCT116
d
OD Value
DLD1
e
NC+DMSO
Fenofibrate (20μM)
Fenofibrate (50μM)
pcDNA3.1-YTHDF3
pcDNA3.1-YTHDF3 +Fenofibrate(50μM)
HCT116
DLD1
f
HCT116
DLD1
Cell invasion (Fold change)
ns
g
WT
KO-YTHDF3
KO-YTHDF3+pcDNA3.1-YTHDF3
KO-YTHDF3+KO-PPARα
HCT116
h
DLD1
i
NC
KO-YTHDF3
KO-YTHDF3 + pcDNA3.1-YTHDF3
KO-YTHDF3 + KO-PPARα
HCT116
DLD1
j
Cell invasion (Fold change)

Figure S12. PPARα activation or knockout rescues YTHDF3-driven invasion, establishing it as an essential downstream effector

a-b. YTHDF3 KO or PPARα-KO CRC cells were generated using CRISPR–Cas9.

c-d. Rescue assays of CCK8 in fenofibrate-treated CRC cells.

e-f. Rescue assays of Transwell assays in fenofibrate-treated CRC cells.

g-h. Rescue assays of CCK8 in CRC cells with re-expressed YTHDF3 or knockout of PPARα in YTHDF3-KO cells.

i-j. Rescue assays of Transwell in CRC cells with re-expressed YTHDF3 or knockout of PPARα in YTHDF3-KO cells.

Fig. S13

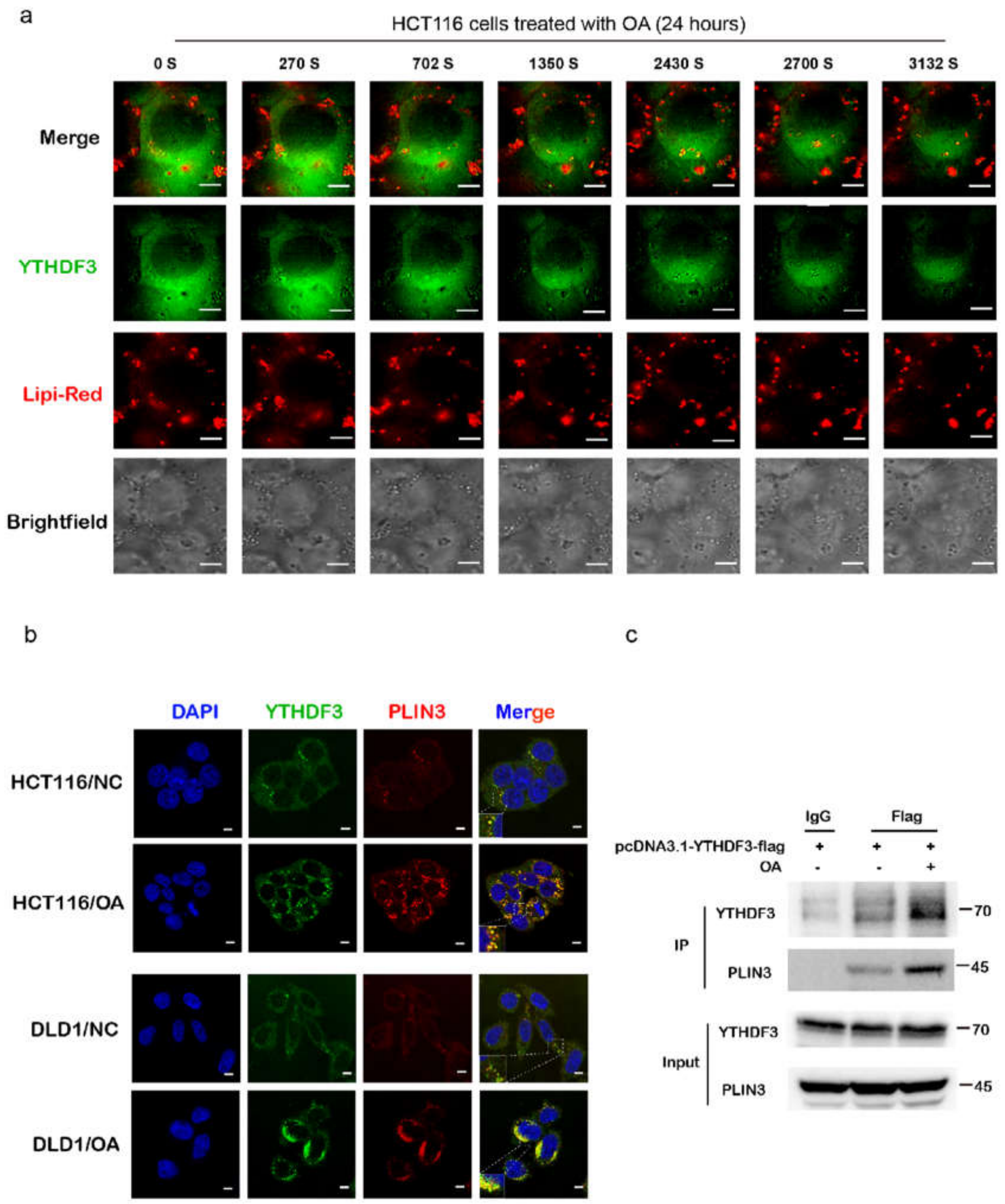

Figure S13. Lipids deposition enhances the co-localization of YTHDF3 and lipid droplets

a. The an ultra-high-resolution microscope assay showed YTHDF3 protein rapidly underwent fusion and fission events in response to the movement of lipid droplets.

b. Confocal immunofluorescence assays revealed the co-localization of YTHDF3 with PLIN3 in CRC cells.

c. CO-IP experiments showed the interaction between YTHDF3 and PLIN3 proteins in CRC cells.

Fig. S14

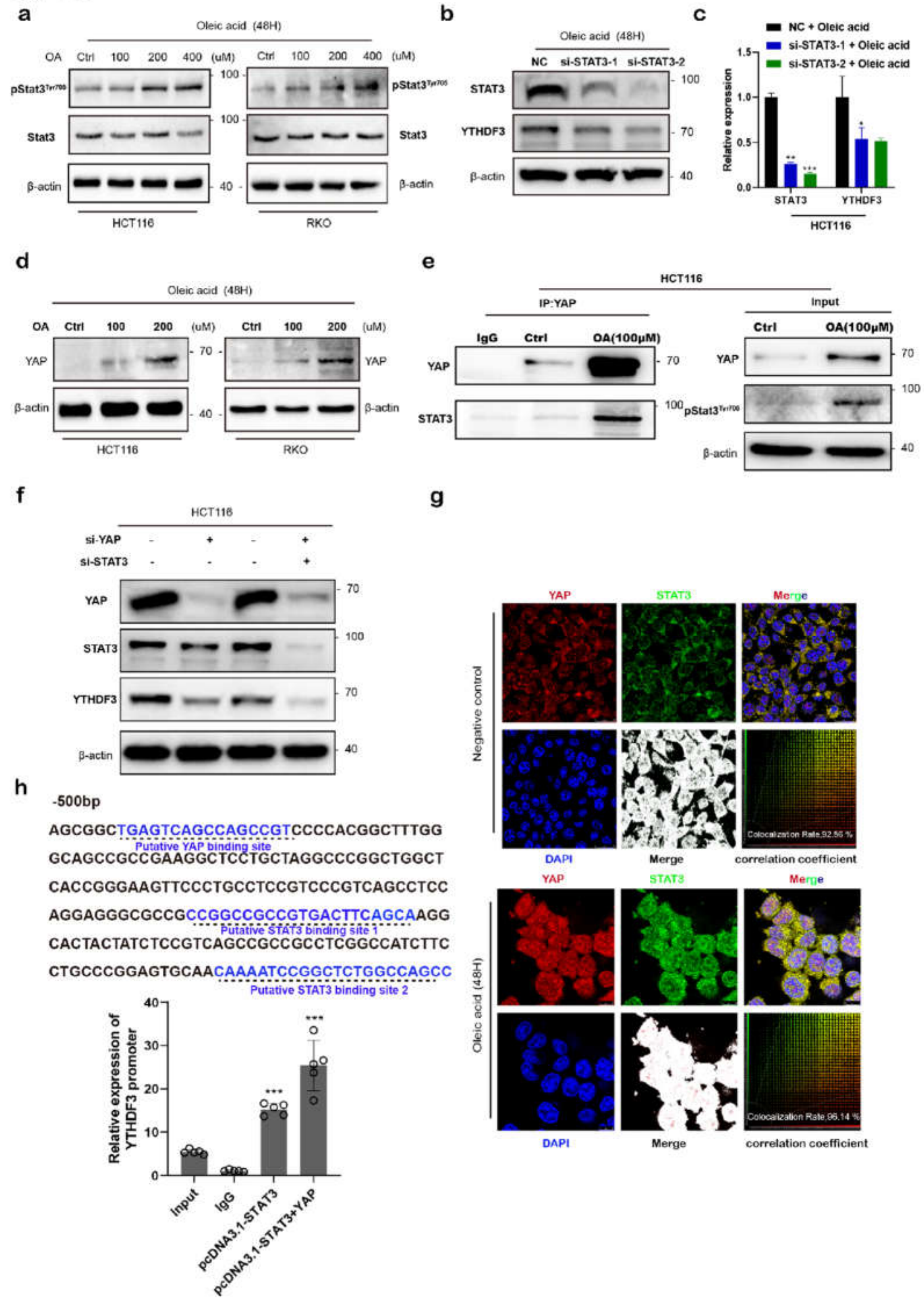

Figure S14. Lipid accumulation enhances STAT3-YAP interaction and facilitates

YTHDF3 expression

a. Western blotting showed the expression level of pSTAT3 ^{Tyr705} in CRC cells treated with various concentrations of OA.

b-c. Western blot (b) and qRT-PCR (c) of indicated markers in STAT3 knockdown CRC cells.

d. Western blotting showed OA promotes the expression of YAP in CRC cells.

e. Co-IP analysis indicated interaction between YAP and STAT3 in CRC cells treated with OA.

f. Western blotting showed the expression of indicated proteins in CRC cells transfected with YAP-specific siRNA or co-transfected with STAT3.

g. Immunofluorescence of STAT3, YAP in HCT116 cells treated with OA for 48 hours.

h. Potential YAP and STAT3 binding sites in the human YTHDF3 promoter (upper panel). Binding of YAP and STAT3 to the YTHDF3 promoter was studied by ChIP assay. And the coprecipitated DNA was subjected for analysis of YTHDF3 by qRT-PCR (bottom panel). Experiments were performed in triplicate, and data are presented as mean ± SD. ***$P < 0.001$.

fig. S15

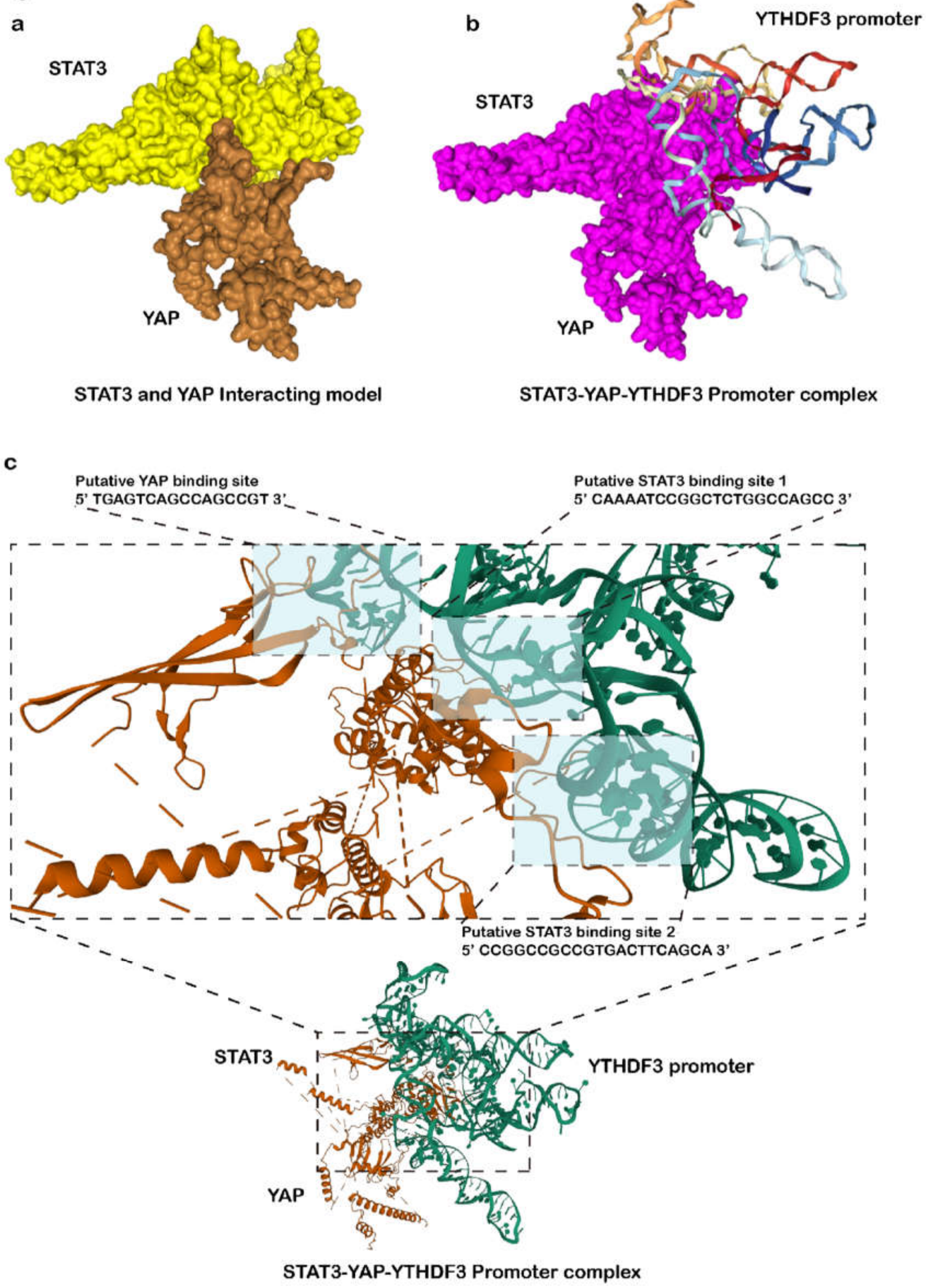

Figure S15. Structural analysis of STAT3-YAP-YTHDF3 promoter complexes

a. Structure of the STAT3-YAP interacting complex.

b. Electrostatic potential surface model of the STAT3-YAP association complex and the

binding sites of YTHDF3 transcriptional start site.

c. Structure of the STAT3-YAP-YTHDF3 promoter complexes. The structure of the STAT3-YAP interacting complex was shown in orange cartoon, with YTHDF3 transcriptional start site was in cyan. The binding sites between protein and promoter are indicated using dashed box. The structure figures were produced with HDOCK.

fig. S16

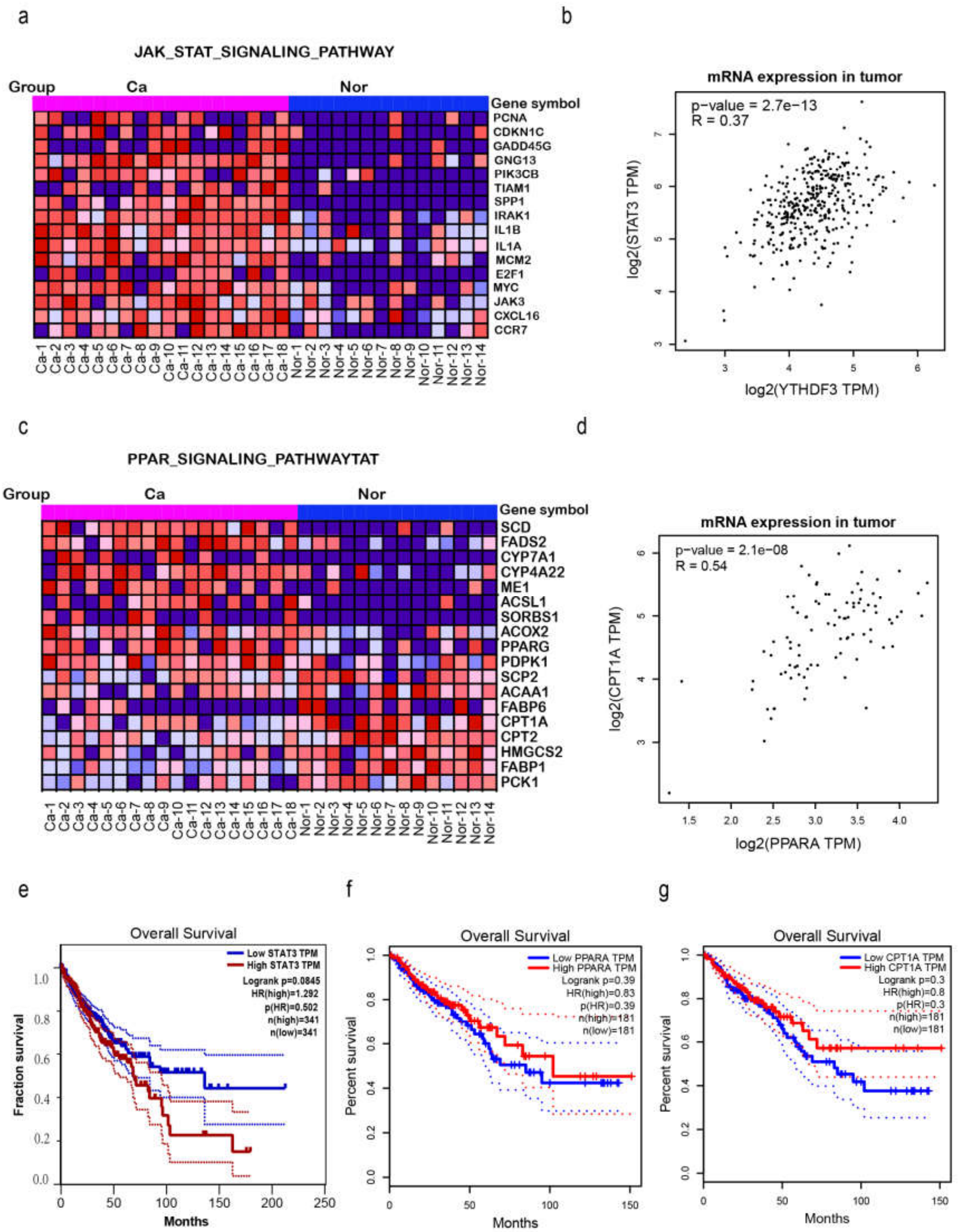

Figure S16. Transcriptome profile of JAK/STAT and PPAR signaling pathway in CRC.

a. Heatmap of the mRNA abundance of JAK/STAT pathway-related genes in CRC tissues (n=18) compared with those in the normal intestinal mucosa (n=14).

b. Correlation analysis was applied between mRNA expressions of STAT3 and YTHDF3 in CRC tissues in TCGA database.

c. The expression levels of PPAR signaling pathway in CRC tissues (n=18) compared with those in the normal intestinal mucosa (n=14).

d. Correlation analysis was applied between mRNA expressions of PPARα and CPT1A in CRC tissues in TCGA database.

e-g. Kaplan-Meier analysis of overall survival (OS) of CRC patients with STAT3, PPARα and CPT1A.

KEY RESOURCES TABLE

REAGENT or RESOURCE	SOURCE	IDENTIFIER
**Antibodies**		
Anti-YTHDF3 antibody	abcam	Cat# ab220161
Anti-Ki67 antibody	abcam	Cat# ab15580
Anti-6X His tag® antibody	abcam	Cat# ab18184
Anti-FLAG® tag antibody	abcam	Cat# ab205606
Anti-PPAR alpha antibody	abcam	Cat# ab227074
Anti-CPT1A antibody	abcam	Cat# ab234111
YAP (D8H1X) XP® rabbit mAb	Cell Signaling Technology	Cat# 14074
Phospho-STAT3 (Tyr705) Antibody	Affinity Biosciences	Cat# AF3293
Stat3 (124H6) Mouse mAb	Cell Signaling Technology	Cat# 9139
Beta Actin Monoclonal antibody	Proteintech	Cat# 66009-1-Ig
**Bacterial and virus strains**		
DH5alpha Competent E. coli	TIANGEN	Cat# CB101
pCDH-MSCV-MCS-EF1-GFP-puro-YTHDF3-3XFLAG plasmid	Shanghai Integrated Biotech Solutions Co.,Ltd	Cat# Y99022
pET28a-SUMO-DogTag plasmid	Addgene	Cat# 105629
LV2(U6/Puro)	GenePharma	N/A
LV6(EF-1aF/Puro)	GenePharma	N/A
**Biological samples**		
Formalin-fixed paraffin-embedded (FFPE) CRC tissues and adjacent normal tissues	Department of Pathology at Sun Yat-sen Memorial Hospital, Sun Yat-sen University	N/A
**Chemicals, peptides, and recombinant proteins**		
Actinomycin D	MedChemExpress	Cat# HY-17559
Azoxymethane	Sigma	Cat# A5486
Dextran sulfate sodium	Sigma	Cat# 9011-18-1
**Critical commercial assays**		
Fatty Acid Oxidation Assay	AssayGenie	Cat# BR00001
Seahorse XF Real-Time ATP Rate Assay Kit	Agilent	Cat# 103592-100
Human TG (triglyceride) ELISA kit	MEIMIAN	Cat# BWM-MM-2101H1
Phospholipid ELISA kit	MEIMIAN	Cat# MM-2491H1
EpiQuik CUT&RUN m6A RNA Enrichment Kit	A&D Technology Corporation	Cat# A-P-9018
Bezafibrate	Selleck	Cat# S4159
**Deposited data**		

MeRIP-seq Raw data	This paper	GSE129716
Transcriptome-seq Raw data	This paper	The data will become public when this manuscript is published online.
Lipidomics-seq Raw data	This paper	The data will become public when this manuscript is published online.
**Experimental models: Cell lines**		
Human: DLD1	Cellcook Biotech Co.,Ltd.	Cat# CC0507
Human: LOVO	Cellcook Biotech Co.,Ltd.	Cat# CC0509
Human: SW480	Cellcook Biotech Co.,Ltd.	Cat# CC0505
Human: SW620	Cellcook Biotech Co.,Ltd.	Cat# CC0503
Human: LS174T	Cellcook Biotech Co.,Ltd.	Cat# CC0513
Human: HCT116	Cellcook Biotech Co.,Ltd.	Cat# CC0506
Human: RKO	Cellcook Biotech Co.,Ltd.	Cat# CC0501
Human: HT29	Cellcook Biotech Co.,Ltd.	Cat# CC0504
**Experimental models: Organisms/strains**		
Mice: BALB/c	Guangdong Medical Laboratory Animal Center	N/A
C57BL/6-Ythdf3 knockout mice	Cyagen	Cat# S-KO-06231
**Oligonucleotides**		
YTHDF3-Homo-1: GCAATGATACTTTGAGTAAGG	GenePharma	N/A
YTHDF3-Homo-2: GCAGTGGTATGACTAGCATTG	GenePharma	N/A
siRNA target: YAP; F: GGUGAUACUAUCAACCAAAGC; R: UUUGGUUGAUAGUAUCACCUG	Shanghai Integrated Biotech Solutions Co.,Ltd	N/A
siRNA target: STAT3; F: GCAGCAGCUGAACAACAUGUTT; R: ACAUGUUGUUCAGCUGCUGCTT	Shanghai Integrated Biotech Solutions Co.,Ltd	N/A
YTHDF3-F: TCAGAGTAACAGCTATCCACCA	The Beijing Genomics Institute	N/A

YTHDF3-R: GGTTGTCAGATATGGCATAGGCT	The Beijing Genomics Institute	N/A
PPARa-F: TTCGCAATCCATCGGCGAG	The Beijing Genomics Institute	N/A
PPARa-R: CCACAGGATAAGTCACCGAGG	The Beijing Genomics Institute	N/A
CPT1A-F: TCCAGTTGGCTTATCGTGGTG	The Beijing Genomics Institute	N/A
CPT1A-R: TCCAGAGTCCGATTGATTTTTGC	The Beijing Genomics Institute	N/A
STAT3-F: ACCAGCAGTATAGCCGCTTC	The Beijing Genomics Institute	N/A
STAT3-R: GCCACAATCCGGGCAATCT	The Beijing Genomics Institute	N/A
**Recombinant DNA**		
pCDH-CMV-MCS-EF1-Puro-YTHDF3	Shanghai Integrated Biotech Solutions Co.,Ltd	Cat. # CD510B-1
pcDNA3.1（+）-YAP1-6xhis	Shanghai Integrated Biotech Solutions Co.,Ltd	Lot No. M25539
pcDNA3.1（+）- STAT3-3xflag	Shanghai Integrated Biotech Solutions Co.,Ltd	Lot No. M25562
**Software and algorithms**		
ImageJ	Schneider et al., 2012	https://imagej.nih.gov/ij/
GraphPad Prism	Prism - graphpad.com	https://www.graphpad.com/company/
